# Metabolic reprogramming in colorectal cancer: Mechanisms and therapeutic insights

**DOI:** 10.1016/j.gendis.2026.102054

**Published:** 2026-01-28

**Authors:** Wen Xiao, Dan Tan, Wen Zeng, Xiaoming Nie, Mingliang Wang

**Affiliations:** a Department of General Surgery, Ruijin Hospital, Shanghai Jiao Tong University School of Medicine, Shanghai 200025, China; b Department of Surgical Oncology, Ganzhou Cancer Hospital, Gannan Medical University, Ganzhou, Jiangxi 341000, China

**Keywords:** Colorectal cancer, Drug resistance, Metabolic reprogramming, Metastasis, Warburg effect

## Abstract

Metabolic reprogramming is an important factor contributing to tumor medication resistance and recurrence in cancer cells. There is ample proof that metabolic remodeling is vital in colorectal cancer’s genesis, progression, and resistance to treatment. The molecular processes behind changes in metabolism in colorectal cancer are methodically examined in this review, including its interactions with the tumor microenvironment and key signaling pathways, and its potential clinical translational value. Investigating the metabolic characteristics of colorectal cancer and identifying novel metabolic biomarkers will provide essential support for early diagnosis and personalized therapy.

## Introduction

Colorectal cancer (CRC) accounts for 9.6% of the global cancer burden, rendering it the third most frequent cancer worldwide, following lung carcinoma and breast carcinoma. It is also the second most common cause of cancer deaths, responsible for approximately 9.3% of all cancer-related mortality rates.^1^ At the time of diagnosis, approximately one-fifth of CRC patients already have distant metastasis, while another quarter of them are initially diagnosed with confined cancer that might eventually progress to metastases.^2^ At this stage, surgical excision, chemotherapy, and radiation are the primary therapeutic choices for CRC. Unfortunately, the resistance, recurrence, and metastasis following radical resection are major causes of cancer-related mortality.^3^^,^^4^ Since most CRC patients present with symptoms at an advanced stage, exploring novel biomarkers is of great significance for improving early screening methods, optimizing personalized treatment, and reducing mortality.^5^ Metabolism encompasses a web of biological processes that transform nutrients into tiny molecules, commonly referred to as metabolites.^6^^,^^7^ By means of a series of metabolic transformations and the metabolites that are produced, cellular systems generate the energy, reducing equivalents, and macromolecules essential for cellular homeostasis and viability. Mutations of oncogenes and tumor suppressor genes induce metabolic reprogramming in cancer, enhancing nutrient uptake to sustain energy production and biosynthetic pathways.^7^^,^^8^ Therefore, understanding the intricate interaction between metabolic reprogramming and CRC is crucial for improving the treatment approaches for CRC. Here, we summarize the latest research on several types of metabolic reprogramming, including the metabolism of glucose, lipids, and amino acids, as well as their relationships with immunity, the tumor microenvironment, and drug resistance. We also introduce the signaling pathways and key mutations associated with metabolic reprogramming in CRC. Finally, we conclude with a summary of the metabolic remodeling associated with distant metastases in CRC.

## The basic mechanisms of metabolic reprogramming

### Glucose metabolic reprogramming

#### *Aerobic glycolysis in CRC*

Glucose metabolic reprogramming constitutes a defining characteristic of malignancies and mediates colorectal carcinogenesis. One of the most prominent metabolic features of cancer cells is the preference for aerobic glycolysis, commonly referred to as the Warburg effect, where glucose is converted into lactate even in the presence of oxygen.^9^^,^^10^ Research indicates that the Warburg metabolic phenotype is ubiquitously observed across rapidly dividing cell populations. Beyond cancer cells, other normal proliferating cells in nature, as well as rapidly growing unicellular organisms, also engage in aerobic glycolysis. The majority of healthy cells in tissues where tumor cells arise usually do not use aerobic glycolysis; this mechanism has been seen across a spectrum of malignancies originated deriving from ontogenetically distinct lineages. This suggests that oncogene-driven populations return to a metabolic profile of cells that divide quickly, indicating that aerobic glycolysis provides a specific advantage during proliferation. Some researchers propose that this metabolic reprogramming serves primarily to sustain elevated amounts of glycolytic intermediates, which include glucose-6-phosphate, 3-phosphoglycerate, and dihydroxyacetone phosphate, which are then utilized to synthesize essential building blocks for biosynthesis, thereby supporting cellular synthetic reactions.^11^ In cancer cells, around 85% of glucose is diverted to aerobic glycolysis, even with sufficient oxygen and glucose supply, with only about 5% entering the mitochondria for oxidative phosphorylation (OXPHOS).^12^^,^^13^ While glycolytic ATP yield per glucose molecule remains inferior to mitochondrial respiration, glycolytic flux achieves a two orders of magnitude acceleration in ATP turnover compared with OXPHOS.^14^ The ATP yield from glycolysis is relatively low, but it is adequate to fulfill cellular demands. Rapidly dividing cells, such as bacteria (with doubling times spanning from a few minutes to several hours), need ATP to continue growing. In contrast, tumor cells, which have a longer doubling time (measured in days instead of minutes), may primarily need ATP to maintain basic cellular functions rather than for proliferation. Consequently, the ATP produced through glycolysis is adequate to support the development of tumor cells.^11^^,^^15^

In the context of glucose metabolic reprogramming in CRC, several regulatory mechanisms converge to promote glycolysis at the expense of mitochondrial oxidation. Liu et al demonstrated that protein arginine methyltransferase 1 (PRMT1) drives asymmetric dimethylation of phosphoglycerate kinase 1 (PGK1) at R206, enhancing its phosphorylation at S203. This post-translational modification inhibits mitochondrial function and redirects carbon flux toward glycolysis.^16^ Complementing this finding, Jing et al demonstrated that elevated expression of non-SMC condensin II complex subunit D3 (NCAPD3) in colorectal carcinoma is clinically associated with unfavorable outcomes, and mechanistically enhances glycolysis via transcriptional control. Specifically, NCAPD3 up-regulates c-Myc to activate glycolytic enzyme expression and cooperates with E2F1 to suppress pyruvate dehydrogenase (PDH) activity, thereby limiting TCA cycle flux.^17^ At the mitochondrial pyruvate transport level, reduced expression of mitochondrial pyruvate carrier 1 (MPC1), a core component of the MPC complex alongside MPC2, is frequently observed in colorectal adenocarcinoma. Loss of MPC1 restricts pyruvate entry into mitochondria, impairs oxidative metabolism, and promotes stem-like properties, correlating with increased metastatic potential.^18^, ^19^, ^20^ However, metabolomic profiling of human colorectal tumors has shown that OXPHOS remains functional and can operate alongside glycolysis, highlighting the metabolic flexibility of CRC cells. This interaction between mitochondrial inhibition and preserved oxidative capacity demonstrates a critical adaptation: CRC cells do not entirely abandon mitochondrial metabolism, but instead adjust it to support proliferation and maintain metabolic flexibility.^21^ Cancer stem cells illustrate this principle by relying heavily on glycolysis to support self-renewal and generate chemoresistance, while retaining the ability to engage mitochondrial pathways when necessary.^22^^,^^23^ These findings underscore the idea that glycolytic reprogramming in CRC is supported at various molecular levels, from post-translational modifications to transcriptional regulation and mitochondrial transport, and that the balance between glycolysis and mitochondrial function is closely tied to cancer stem cell maintenance and tumor aggressiveness. Clarifying how these regulatory mechanisms operate will be essential for defining the metabolic dependencies of CRC. Such knowledge could guide the design of interventions that exploit context-specific metabolic weaknesses, ultimately offering new opportunities to refine therapeutic strategies for this challenging disease.

**Figure 1:**
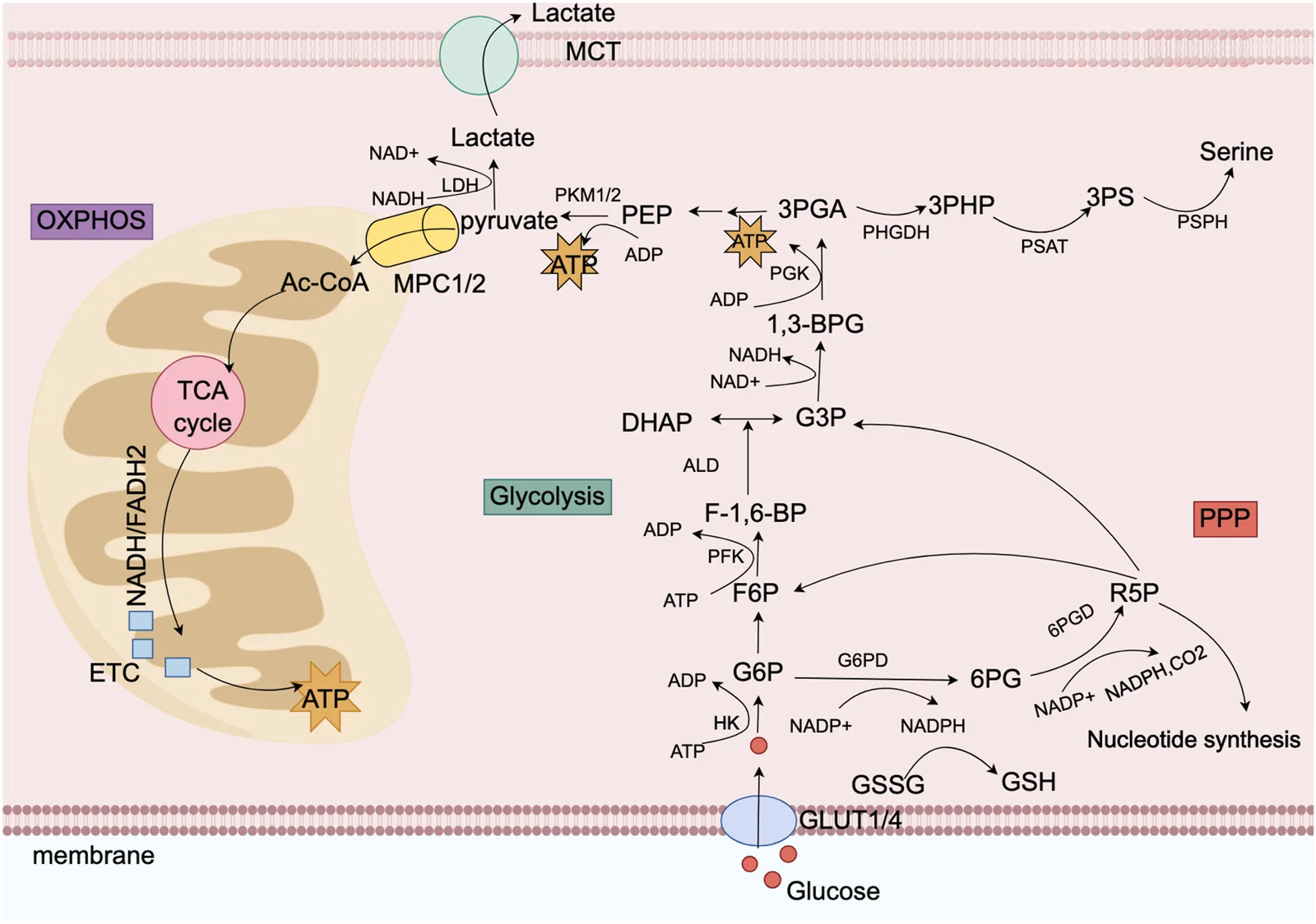
Schematic overview of glucose metabolism in colorectal cancer, including glycolysis, lactate metabolism, OXPHOS, and major branch pathways (PPP, serine synthesis, GSH). Tumor cells preferentially utilize aerobic glycolysis, known as the Warburg effect. Glucose is transported into cells mainly via GLUT transporters and metabolized to pyruvate. Pyruvate is either reduced to lactate by LDH, which is exported through MCTs, or transported into mitochondria by the MPC1/2 to fuel the TCA cycle and OXPHOS. Glycolytic intermediates are diverted into biosynthetic pathways: G6P enters the PPP to R5P for nucleotide synthesis and NADPH for maintaining redox homeostasis through GSH/GSSG cycling, while 3PGA is shunted into the serine biosynthesis pathway (PHGDH–PSAT–PSPH axis) to provide precursors for amino acid and nucleotide synthesis. These reprogrammed metabolic pathways collectively sustain rapid proliferation, redox balance, therapeutic resistance, and immune evasion in colorectal cancer. OXPHOS, oxidative phosphorylation; TCA, tricarboxylic acid cycle; ETC, electron transport chain; LDH, lactate dehydrogenase; MCT, monocarboxylate transporter; MPC, mitochondrial pyruvate carrier; PPP, pentose phosphate pathway; GLUT, glucose transporter; R5P, ribose-5-phosphate; G6P, glucose-6-phosphate; 3PGA, 3-phosphoglycerate; PHGDH, phosphoglycerate dehydrogenase; PSAT, phosphoserine aminotransferase; PSPH, phosphoserine phosphatase; GSH, glutathione; GSSG, oxidized glutathione.

Additionally, pyruvate kinase (PK) M2 isoform facilitates the transition from OXPHOS to aerobic glycolysis. Phosphoenolpyruvate is converted to pyruvate by PKM2, which catalyzes the last stage of glycolysis. Compared with the constitutively active splice variant PKM1, PKM2 is the main variant in cancerous cells.^24^, ^25^, ^26^, ^27^ Notably, heterogeneity in glycolytic dependence among CRC cell lines further underscores the complexity of glucose metabolic reprogramming. Although HCT116, HT29, and SW620 are all colon cancer cell lines, they display heterogeneity in glucose metabolism pathways (such as glycolysis or OXPHOS). Specifically, after PKM1/2 silencing, HCT116 cells can maintain ATP levels and proliferation by increasing OXPHOS, whereas HT29 and SW620 cells show a higher sensitivity in proliferation rates to PKM1/2 silencing, suggesting a greater metabolic dependency on PK activity in these cells.^26^ Interestingly, this metabolic divergence also translates into differential drug responses. Ginés et al demonstrated that PKM2 expression and subcellular localization correlate with oxaliplatin sensitivity: silencing PKM2 induces resistance in HT29 and SW480 cells but increases sensitivity in HCT116 cells. Mechanistically, this discrepancy was linked to differential regulation of the pro-apoptotic gene BMF rather than p53 status, highlighting that the genetic and molecular context shapes the therapeutic outcome. Holistically, mechanistic insights reveal that PKM2 contributes not only to glycolytic flux but also to chemosensitivity through non-metabolic functions, and that metabolic heterogeneity among CRC cell lines critically influences their drug response profiles.^28^ At first glance, the observation that PKM1/2 knockdown impairs proliferation in glycolysis-dependent HT29 cells but enhances oxaliplatin resistance in the same line may appear contradictory. However, this discrepancy highlights the multifaceted role of PKM2, functioning both as a mediator of glycolytic metabolism and a context-sensitive regulator of *pro-apoptotic molecules* (*e.g.*, BMF). These findings underscore that metabolic heterogeneity among CRC cell lines translates into distinct drug response profiles, emphasizing the need for integrative analyses that consider both metabolic and non-metabolic functions of PKM2. Beyond PKM2, other glycolytic branch-point enzymes also modulate CRC metabolism and therapeutic response. For example, phosphoglycerate dehydrogenase (PHGDH), which diverts 3-phosphoglycerate (3-PGA) into the serine biosynthesis pathway, was mechanistically demonstrated to stimulate tumor growth. It is worth noting that, under glucose-limited conditions, PHGDH interacts with p53 to promote tumor cell death rather than supporting serine synthesis, providing a mechanistic rationale for dietary or fasting-based interventions in cancer therapy^29^ (Fig. 1).

Within the neoplastic microenvironment, regulatory T cells (Tregs) undergo distinct metabolic reprogramming to preserve their immunosuppressive capacity. Loss of the transcription factor MondoA has been shown to shift Tregs toward a glycolytic, Th17-like phenotype, thereby aggravating interleukin-17A (IL-17A)-driven dysfunction of CD8^+^ T cells and fostering an inflammatory CRC milieu. Therapeutically, constraining this metabolic plasticity in Tregs may offer a means to reinstate effective anti-tumor immunity.^30^ Moreover, CD8^+^ T-cell impairment can also result from nutrient competition, as effector lymphocytes are frequently outcompeted by both tumor cells and other immunosuppressive cell populations within the metabolic niche.^31^, ^32^, ^33^ A recent study, however, indicates that brown adipose tissue, similar to tumor tissue, exhibits high glucose utilization. Activating brown adipose tissue through exposure to low temperatures, glucose required by the tumor can be deprived, thereby inhibiting tumor growth.^34^ While the brown adipose tissue study is not CRC-specific, when considered together with CRC-related findings on Treg plasticity and nutrient competition, they provide broader perspectives on how systemic and local metabolic pathways can be leveraged to regulate tumor progression and immune evasion.

#### *Lactate metabolism in CRC*

The enzyme lactate dehydrogenase (LDH), which is highly expressed in numerous proliferating cells in mammals, is responsible for converting pyruvate to lactate.^35^ Despite significant advancements in CRC therapy, the reaction frequency remains suboptimal, and the benefits of 5-fluorouracil (5-FU)-based treatments are often undermined by the emergence of chemoresistance. Therefore, Dong et al investigated the metabolism of 5-FU-resistant cells and found elevated lactate levels in subcutaneous tumors formed by these cells, along with significant up-regulation of monocarboxylate transporters (MCT1 and MCT4). Notably, 5-FU-resistant cells exhibited increased sensitivity to syrosingopine, a selective MCT4 inhibitor, and low MCT4 expression enhanced their sensitivity to 5-FU. Importantly, the study revealed 5-FU resistance in CRC is closely linked to glucose metabolic rewiring driven by hypoxia-inducible factor-1 alpha (HIF-1α) signaling. Silencing HIF-1α resulted in the down-regulation of its downstream targets, including LDH-A and MCT4. In nude mouse xenograft models, inhibition of HIF-1α significantly restored sensitivity to 5-FU and suppressed tumor progression. Furthermore, *in vivo* treatment with the HIF-1α inhibitor IDF-11774 produced similar effects to HIF-1α knockdown, underscoring its translational promise for molecularly targeted intervention.^36^ Since LDH-A and MCT4 are downstream effectors of HIF-1α, these findings also imply that inhibiting LDH and MCT4 may be effective *in vivo*. Although *in vivo* studies of syrosingopine in CRC are currently lacking, preclinical research in breast cancer models using liposome-encapsulated lonidamine and syrosingopine (L@S/L) has demonstrated effective modulation of tumor microenvironment pH and alleviation of immunosuppression.^37^ Therefore, applying similar strategies in CRC may hold promising exploratory value. LDH-A has also been implicated in regulating DNA mismatch repair. Although immune checkpoint inhibitors are effective in tumors with deficient mismatch repair (dMMR), more than 80% of CRC patients exhibit proficient mismatch repair (pMMR). Recent studies have uncovered seemingly divergent roles of lactate metabolism in this context. On one hand, Candida tropicalis has been shown to promote oxaliplatin resistance in CRC xenograft models by enhancing glycolytic flux and elevating lactate production. Lactate in turn suppresses the expression of MLH1 and MSH2 via the GPR81–cAMP–PKA–CREB signaling axis, thereby compromising MMR function and promoting oxaliplatin resistance. Inhibition of lactate production restored MMR protein expression and resensitized tumors to oxaliplatin.^38^ On the other hand, Zhang et al found that LDH-A activity positively regulated MMR protein expression in CRC cells, sustaining tumor proliferation in both dMMR and pMMR backgrounds. The same group further linked LDH-A to the up-regulation of stemness-associated genes such as Oct4 and Sox2, thereby contributing to an immunosuppressive “cold” tumor phenotype.^39^ These contrasting observations highlight that lactate metabolism may influence MMR stability in a compartment- and context-dependent manner. Clarifying these distinct mechanisms shall prove indispensable in crafting rational mechanism-based interventions. In particular, dissecting how lactate flux and LDH-A activity integrate with stemness programs and immune evasion could inform the design of combination regimens that pair metabolic inhibitors with chemotherapy or immunotherapy. Future work should also assess whether targeting lactate signaling pathways or selectively modulating LDH-A activity can differentially sensitize pMMR and dMMR CRC subtypes to existing treatments.

Emerging investigations propose that lactate also serves as a significant carbon source in aerobic environments, which is oxidized in mitochondria. A landmark 2017 study by Faubert et al demonstrated that human non-small cell lung cancer cells oxidize circulating lactate as a major fuel for the TCA cycle, even exceeding glucose under certain conditions. By combining U–13C lactate tracing with CRISPR/Cas9 knockout of MCT1, they showed that lactate uptake depends critically on MCT1.^40^ In the CRC field, more recent evidence also supports a role for lactate as a TCA substrate. For example, Montal et al used stable isotope tracing and showed that CRC cells incorporated U–13C lactate into TCA cycle intermediates (*e.g.*, citrate, α-ketoglutarate), particularly under nutrient deprivation, and that inhibiting phosphoenolpyruvate carboxykinase (PEPCK) reduced this lactate-fueled TCA flux and tumor growth.^41^ Moreover, in cetuximab-resistant CRC models, pharmacological inhibition of MCT1 with the preclinical tool compound AR-C155858 reduced lactate uptake and oxidation, suppressing clonal expansion *ex vivo* and xenograft progression in immunocompromised hosts, thereby highlighting MCT1-dependent lactate metabolism as a potential therapeutic vulnerability.^42^ Taken as a whole, the evidence indicates that lactate metabolism functions as an adaptive strategy rather than a passive byproduct, thereby exposing potential metabolic vulnerabilities that may be therapeutically exploited in CRC. Additionally, some research has shown that Tregs strongly produce MCT1 in order to absorb lactate from the tumor microenvironment for gluconeogenesis, consequently reinforcing immune suppression. Lactate uptake by Tregs up-regulates their programmed cell death 1 (PD-1) expression, reducing PD-1 availability on CD8^+^ T lymphocytes and impairing PD-1 blockade efficacy.^43^^,^^44^ Disrupting the metabolic symbiosis between Tregs and cytotoxic T cells may improve the effectiveness of immunotherapy, according to these researchers.

Beyond these classical roles, lactate has recently been recognized as a regulatory factor through protein lactylation, particularly in the glycolysis-enriched tumor microenvironment. For example, KAT8 has been shown to promote abnormal lysine lactylation, thereby facilitating CRC progression.^45^ Elevated histone lactylation levels in CRC patients have also been reported to enhance the transcription of RUBCNL, which contributes to resistance against bevacizumab therapy.^46^ In addition, GPR37 has been demonstrated to activate the Hippo molecular cascade, thereby increasing glycolysis and lactate accumulation, which subsequently induces histone H3K18 lactylation and promotes CRC liver metastasis.^47^ These findings highlight lactylation as a novel layer of metabolic regulation that links aberrant glycolysis with epigenetic modifications, therapeutic resistance, and metastatic progression in CRC. The latest evidence from Cai et al further revealed that lactate exhibits dual capacity to maintain bioenergetic fluxes while fundamentally orchestrating immunological priming. In immune-trained monocytes, lactate is preferentially utilized as a fuel for the TCA cycle, promoting histone lactylation and enhancing the transcription of immune-related genes. Silencing lactate dehydrogenase A (LDHA) disrupts lactate-driven metabolism and lactylation, resulting in reduced cytokine production.^48^ This study highlights lactate as a central hub in the metabolic, immune, and epigenetic regulation of trained immunity in monocytes. However, the study is limited to basic research and does not address specific disease models or extend to clinical treatment applications. Future studies could explore the role of lactate regulation in various disease contexts, particularly in tumor microenvironments, where it may influence immune evasion mechanisms. Additionally, the therapeutic implications of lactate in immune therapies, particularly in combination with immune checkpoint inhibitors, need to be further explored in clinical settings.

Research on human glioma cells has demonstrated that only 7% of glucose uptake is devoted to macromolecule production, with the remaining 93% going to the synthesis of lactate and alanine; this quantitative partitioning is context-dependent and is provided here as a conceptual example, not a CRC-specific constant. The high flux of glycolysis toward lactate occurs because LDH converts pyruvate to lactate, regenerating NAD^+^ and maintaining the NAD^+^/NADH redox balance. This balance accelerates glycolysis.^35^ Additionally, NAD^+^ is necessary for nucleotide and polypeptide synthesis; thus, the simultaneous manufacture of lactate effectively replenishes NAD^+^, enhancing the incorporation of glucose metabolites into biomass.^11^^,^^35^ Lactate production may indirectly support tumor cell infiltration and survivability while inhibiting anti-cancer T cell immune responses.^49^, ^50^, ^51^ Although these effects promote tumor growth, high lactate excretion is unlikely to be the primary selective pressure driving aerobic glycolysis. Instead, to maintain glycolytic flow, cells preferentially shift toward lactate production to renew NAD^+^. Furthermore, it appears that increased lactate excretion is not the primary cause of cancer cells adopting this metabolic pathway, as many healthy proliferating cells utilize aerobic glycolysis without deriving benefits from lactate.^11^

#### *Lipid metabolism reprogramming*

Lipid molecules are classified into eight groups. Notably, some of them are linked with the progression of CRC.^52^ Tumor cells adopt diverse strategies to obtain lipids and boost lipid oxidation in challenging environments with limited oxygen and nutrients. This abnormal lipid metabolism is known as lipid metabolic reprogramming. Cancer cells utilize abnormal fat metabolism to acquire more constituents for biological membranes and signaling molecules necessary for metastatic growth and invasion. As research into lipid metabolism progresses, an increasing amount of data indicates that lipid metabolism reprogramming is a characteristic of tumors.^53^, ^54^, ^55^, ^56^

#### *Role of CPT1/2 in fatty acid oxidation and tumor progression*

**Figure 2:**
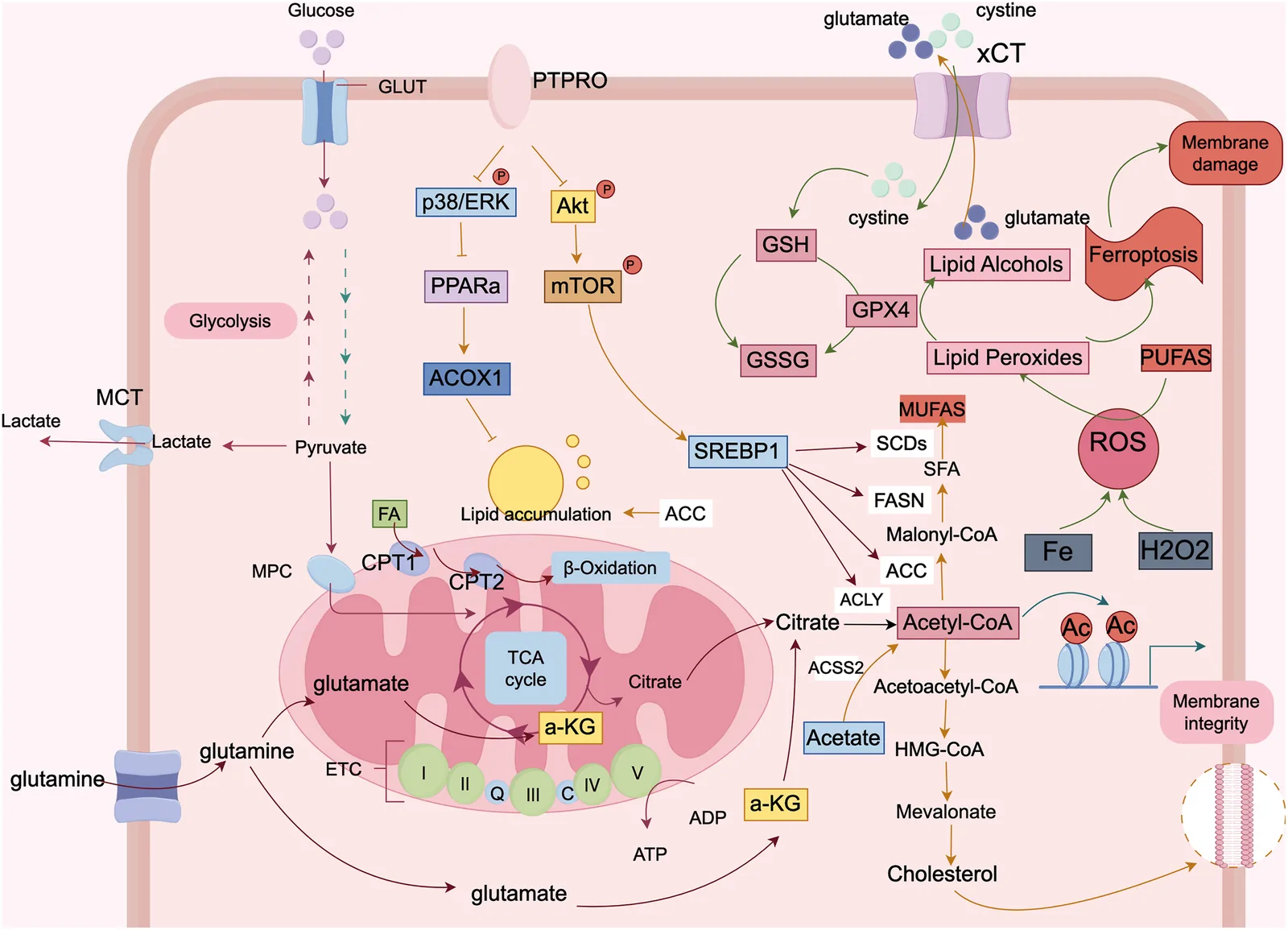
Lipid metabolism pathway and ferroptosis. Colorectal cancer cells adapt to nutrient and oxygen limitations through lipid metabolic reprogramming. Citrate-derived acetyl-CoA, generated via ACLY, serves as the central substrate for fatty acid synthesis and histone acetylation. SREBP1 transcriptionally regulates ACLY, ACC, FASN, and SCD1, thereby promoting *de novo* lipid biosynthesis. Fatty acids can be further oxidized in mitochondria through CPT1/2 to sustain energy production. Alternatively, acetate and glutamine-derived α-KG act as carbon sources for lipid synthesis, particularly under hypoxia. Dysregulated lipid metabolism enhances membrane biosynthesis, supports redox balance, and drives tumor growth and ferroptosis resistance in colorectal cancer. ACLY, ATP-citrate lyase; ACC, acetyl-CoA carboxylase; FASN, fatty acid synthase; SCD1, stearoyl-CoA desaturase 1; CPT1/2, carnitine palmitoyl transferase 1/2; SREBP1, sterol regulatory element-binding protein 1; α-KG, α-ketoglutarate; ROS, reactive oxygen species; GPX4, glutathione peroxidase 4.

Carnitine palmitoyl transferase (CPT) facilitates the delivery of long-chain fatty acids (FAs) to the mitochondrial matrix, including CPT1 and CPT2 (Fig. 2). In CRC, CPT1A expression is markedly up-regulated when tumor cells are exposed to adipocytes or FAs, particularly in lesions invading adipose-rich niches. CPT1A-dependent fatty acid oxidation (FAO) not only protects tumor cells from nutrient stress but also maintains cancer stemness by promoting β-catenin acetylation and nuclear translocation, thereby enhancing organoid formation and xenograft initiation.^57^ This evidence underscores a tumor-promoting role for CPT1A, linking adipocyte interactions and lipid-rich microenvironments to CRC progression through FAO-driven stemness programs. The role of CPT2, however, is more complex and context-dependent. While CPT2 is indispensable for the growth of many malignancies,^58^^,^^59^ its function in CRC appears heterogeneous. Earlier evidence suggested that CPT2 down-regulation enhances glycolysis, stem-like traits, and resistance to oxaliplatin, implying a tumor-suppressive function under certain metabolic conditions.^60^ In contrast, a more recent report demonstrated that solute carrier family 44 member 4 (SLC44A2) acts as a tumor suppressor by promoting MUL1-mediated CPT2 degradation, thereby attenuating mitochondrial FAO and limiting CRC growth and metastasis.^61^ At first glance, these findings appear contradictory; however, they more likely reflect differences in experimental emphasis and cellular context. The 2021 study mainly addressed how loss of CPT2 reshapes glycolysis and drug resistance programs, whereas the 2025 investigation linked SLC44A2 to mitochondrial FAO modulation and energy/redox balance. Together, these observations underscore that the role of CPT2 in CRC is not uniform but rather highly context-dependent, influenced by metabolic compartmentalization and interacting signaling pathways. Future studies should aim to clarify these discrepancies by evaluating CPT2 expression and FAO flux in parallel with stemness markers, redox status, and therapy responses within the same CRC models, as well as validating these findings in patient-derived samples. Such integrative approaches will be essential to determine whether targeting CPT2 or its upstream regulators, such as SLC44A2, could represent viable therapeutic strategies in CRC.

#### *Rewiring of carbon flux and lipid biosynthetic networks in CRC*

In an environment rich in nutrients, for lipid synthesis in mammalian cells, mitochondrial citrate is the principal source of acetyl-CoA. However, tumor cells preferentially undergo aerobic glycolysis, converting pyruvate to lactate, thereby restricting the quantity of pyruvate going into the Krebs cycle. As a result, citrate production and acetyl-CoA synthesis via ATP-citrate lyase (ACLY) in the cytoplasm are reduced. Despite this, emerging evidence posits that acetate could serve as a compensatory substrate for acetyl-CoA generation in cancer cells, thereby supporting enhanced lipid synthesis.^62^, ^63^, ^64^, ^65^ Mitochondrial citrate, produced in the Krebs cycle, is exported to the cytoplasm by mitochondrial citrate carrier (CIC, encoded by SLC25A1), where it is essential for the production of *de novo* FAs. Research has demonstrated elevated CIC levels across multiple cancer lineages, with its function being essential for progression. For instance, according to a study by Ozkaya et al, blocking CIC lowers breast cancer survival.^66^^,^^67^ Emerging evidence indicates the pathological contribution of SLC25A1 in CRC. Yang et al demonstrated that SLC25A1 is markedly overexpressed in CRC compared with adjacent normal tissues, and its high expression predicts unfavorable clinical outcomes. Functional assays revealed that silencing SLC25A1 suppressed CRC proliferation and colony formation, supporting the notion that CIC drives tumor growth via citrate export and lipid biosynthesis. Interestingly, SLC25A1 depletion did not affect invasion or metastasis, suggesting that its contribution may be more restricted to sustaining proliferation rather than driving dissemination. Notably, beyond its canonical role in citrate efflux, CIC can also operate in reverse to transport cytosolic citrate back into mitochondria under stress conditions, potentially reinforcing OXPHOS.^68^ This bidirectional capacity underscores the metabolic plasticity inherent to CRC cells, enabling their capacity to adjust between anabolic demands and oxidative requirements. While small-molecule CIC inhibitors have been described, their potential in CRC remains largely unexplored. Future studies should investigate how CIC integrates with other metabolic pathways and whether targeting CIC could be combined with existing therapies to achieve better disease control.

Downstream of CIC-exported citrate, ACLY, the initial key enzyme, is raised among pan-cancer entities, exemplifying colorectal cancer,^69^ glioblastoma,^70^ breast cancer,^71^ and ovarian cancer.^72^ Overexpression of ACLY promotes tumor growth, while its inhibition or knockout suppresses cell proliferation.^73^, ^74^, ^75^, ^76^ ACLY serves as a critical nexus between glucose and lipid metabolism through the generation of cytosolic acetyl-CoA, and its relevance now extends beyond tumor cell intrinsic functions. Recent evidence demonstrated that pharmacological inhibition of ACLY can overcome resistance to immune checkpoint blockade by promoting peroxidation of polyunsaturated FAs and activating the cGAS–STING pathway, highlighting ACLY as a metabolic target with immunomodulatory potential in solid tumors.^77^ Complementing these findings, another study revealed that exhausted CD8^+^ T cells (TEX) maintain citrate-dependent histone acetylation through an ACLY–KAT2A axis while down-regulating acetate–ACSS2–p300-mediated acetylation, thereby reinforcing the transcriptional program of exhaustion. ACLY inhibition disrupts this process, prevents TEX differentiation, and enhances effector T cell responses. Importantly, these results are validated not only in melanoma but also in MC38, a microsatellite instability-like CRC model, underscoring the translational relevance to CRC immunology.^78^ Nevertheless, it should be emphasized that MC38 represents a microsatellite instability (MSI)-high phenotype, whereas the majority of clinical CRC cases are microsatellite stable (MSS), where immune checkpoint blockade remains less effective. Thus, while current findings suggest that ACLY inhibition may simultaneously restrain tumor growth and remodel T cell immunity, further studies in MSS CRC models and patient-derived samples are needed to establish its therapeutic utility in this more prevalent clinical context.

Acetyl-CoA carboxylase (ACC) is another essential process in the production of FAs. ACC1 is primarily involved in the anabolism of FAs, while ACC2 regulates FA oxidation.^79^ AMP-activated protein kinase (AMPK), activated by antioncogene LKB1, inhibits ACC1 by phosphorylation, thus reducing FA synthesis.^76^^,^^80^ Beyond lipid metabolism, LKB1 has also been implicated in restraining CRC progression through the AMPK–AKT axis, underscoring its significance as both a metabolic regulator and a candidate intervention node.^81^ In addition to these canonical regulators, malonyl-CoA decarboxylase (MCD) and its negative regulator SIRT4 have been implicated in maintaining lipid metabolic balance by modulating the switch between fatty acid synthesis and oxidation, although their specific roles in CRC remain to be fully defined.^82^

FAs are carboxyl compounds with a long hydrocarbon chain, usually even in number. They are crucial for forming membrane-associated lipids, providing energy substrates, and functioning as secondary messengers in signaling pathways.^83^^,^^84^ A central enzyme in this context is fatty acid synthase (FASN), which synthesizes palmitate. FASN is highly expressed in CRC, according to studies, and that blocking tumor cells’ FA uptake and FASN in cancer-associated fibroblasts (CAFs) can dramatically reduce cell migration.^85^, ^86^, ^87^, ^88^ Beyond its canonical lipogenic role, FASN has been experimentally tied to sustaining stemness and ferroptosis resistance in CRC. Wang et al reported that FASN enhanced stem-like traits and protected tumor cells from ferroptotic stress by suppressing sterol regulatory element-binding protein 2 (SREBP2) activation, thereby linking fatty acid synthesis, cholesterol metabolism, and redox balance.^89^ These findings highlight FASN as a central metabolic regulator in CRC progression. Although preclinical data strongly support FASN as a therapeutic target, clinical translation remains limited. Most FASN inhibitors, including TVB-2640 (denifanstat), are currently under early-phase evaluation for metabolic liver diseases and other solid tumors,^90^^,^^91^ with no CRC-specific trials yet reported. Given the emerging evidence that the FASN–SREBP2 axis orchestrates lipid metabolism, stemness maintenance, and ferroptosis resistance, future efforts should explore how modulation of this pathway may synergize with existing chemotherapy or immunotherapy to improve therapeutic outcomes in CRC (Fig. 2).

#### *Short-chain fatty acids in CRC and their mechanisms*

Short-chain fatty acids (SCFAs) like butyrate (NaB) have been reported to have anti-cancer properties via blocking histone deacetylases.^92^, ^93^, ^94^, ^95^ Wang et al demonstrated that NaB activates mitochondrial OXPHOS, suppresses methionine metabolism, as well as modulating nuclear factor erythroid 2-related factor 2 (NRF2) promoter methylation, inhibiting CRC HCT116 cell proliferation.^94^ In line with this, Bian et al observed that NaB notably elevated Fe^2+^ levels within HCT-116 cells, while also enhancing GSH depletion and inducing lipid peroxidation, thereby driving ferroptosis in CRC cells.^96^ Beyond direct metabolic reprogramming, NaB has also been shown to modulate the tumor–immune interface. Zhang et al found that NaB could down-regulate PD-L1 expression in CRC cells and enhance CD8^+^ T cell infiltration, thereby synergistically inhibiting tumor growth.^95^ Similar to the above, Yan et al investigated the role of berberine in an AOM/DSS-induced colitis-associated cancer model and demonstrated that its protective effect was microbiota-dependent. Oral administration of berberine reshaped gut microbial composition, enriched SCFA-producing taxa (*e.g.*, Prevotellaceae, Alloprevotella), and significantly elevated fecal acetate, propionate, and butyrate levels, while reducing LPS concentrations. These microbial and metabolic changes coincided with up-regulation of barrier proteins (Occludin, ZO-1) and inhibition of the toll-like receptor 4 (TLR4)–nuclear factor-kappa B (NF-κB)–IL-6–signal transducer and activator of transcription 3 (STAT3) axis. Moreover, intestinal flora engraftment with specimens from berberine-treated mice reproduced the protective phenotype. Collectively, this study suggests that remodeling of the gut microbiota and increased SCFA production are associated with reduced inflammation-driven colorectal carcinogenesis. Nevertheless, its reliance on the AOM/DSS model and focus on total SCFA levels, rather than single metabolites, limits causal interpretation.^97^ Mechanistically, these results position the pleiotropic functions exerted by SCFAs within the CRC landscape, spanning direct metabolic regulation, immune modulation, and microbiota-mediated pathways, and emphasize their potential as therapeutic targets. However, current evidence is largely preclinical and model-dependent, with limited validation in clinical settings, underscoring the need for integrative studies that bridge mechanistic insights with translational applications.

#### *Alternative lipid anabolic pathways and their functional implications in CRC*

Under conditions of mitochondrial dysfunction or hypoxia, glutamine-derived α-ketoglutarate (α-KG) can undergo reductive carboxylation to produce citrate, serving as an alternative carbon source for lipid synthesis alongside acetate (Fig. 2). Glutamine transporters, particularly solute carrier family 1 member 5 (SLC1A5), are frequently up-regulated in CRC, facilitating enhanced glutamine uptake to sustain biosynthetic and energetic demands. Through this compensatory pathway, glutamine drives fatty acid biogenesis, thereby maintaining tumor viability in metabolically compromised conditions.^98^, ^99^, ^100^, ^101^ The broader metabolic and immunological implications of glutamine utilization in CRC are discussed in the subsection “Glutamine metabolism and its immunometabolic implications in CRC”.

Building on these adaptive mechanisms, lipid desaturation represents another crucial node of metabolic flexibility in CRC. Stearoyl-CoA desaturase 1 (SCD1) is regulated by SREBP1, a downstream target of mechanistic target of rapamycin complex 1 (mTORC1), and functions as a lipid desaturase converting saturated fatty acids to monounsaturated fatty acids (MUFAs) (Fig. 2). Chen et al demonstrated that aspirin inhibits mTORC1 activity, thereby suppressing SREBP1-mediated MUFA synthesis and promoting ferroptosis in CRC.^102^, ^103^, ^104^, ^105^ Beyond SCD1, fatty acid desaturase 2 (FADS2) has been characterized as an alternative source of unsaturated fatty acids, generating sapienate to maintain membrane integrity and compensate for SCD1 inhibition. This finding explains why blockade of SCD1 alone may be insufficient in certain tumors, including those of hepatic or pulmonary origin.^106^ Furthermore, FADS2 expression is closely linked to mTOR signaling, suggesting that this pathway may represent a broader adaptive mechanism for maintaining lipid homeostasis under metabolic stress.^107^ Although evidence in CRC remains limited, further exploration of FADS2-mediated desaturation may uncover additional vulnerabilities for therapeutic intervention.

Beyond fatty acid metabolism, enzymes involved in steroidogenesis also intersect with lipid metabolic reprogramming in CRC. Aromatase (CYP19A1), a key enzyme responsible for estrogen synthesis, exhibits aberrant up-regulation in colorectal, breast, and gastric cancers. Inhibition of aromatase, particularly in combination with the mTOR inhibitor everolimus, has been shown to extend clinical benefit duration in hormone-sensitive cancers by reducing Ki-67 expression and Treg infiltration while enhancing cytotoxic T cell activity. Liu et al further demonstrated that CYP19A1 blockade enhances PD-1 checkpoint blockade responsiveness in CRC, underscoring the link between lipid metabolism and immune modulation.^108^, ^109^, ^110^, ^111^, ^112^, ^113^

Finally, metabolic adaptation to therapy represents a key driver of treatment resistance in CRC. For instance, bevacizumab, an anti-angiogenic agent used in advanced CRC, may lose efficacy as tumor cells rewire lipid metabolism to compensate for nutrient and oxygen deprivation, thereby maintaining survival under reduced vascular supply.^114^^,^^115^ These findings collectively emphasize that lipid synthesis and desaturation pathways in CRC are not only central to tumor growth and metabolic resilience but also intimately connected to therapeutic response and immune regulation.

#### *Lipid metabolism in the tumor microenvironment and CRC progression*

**Figure 3:**
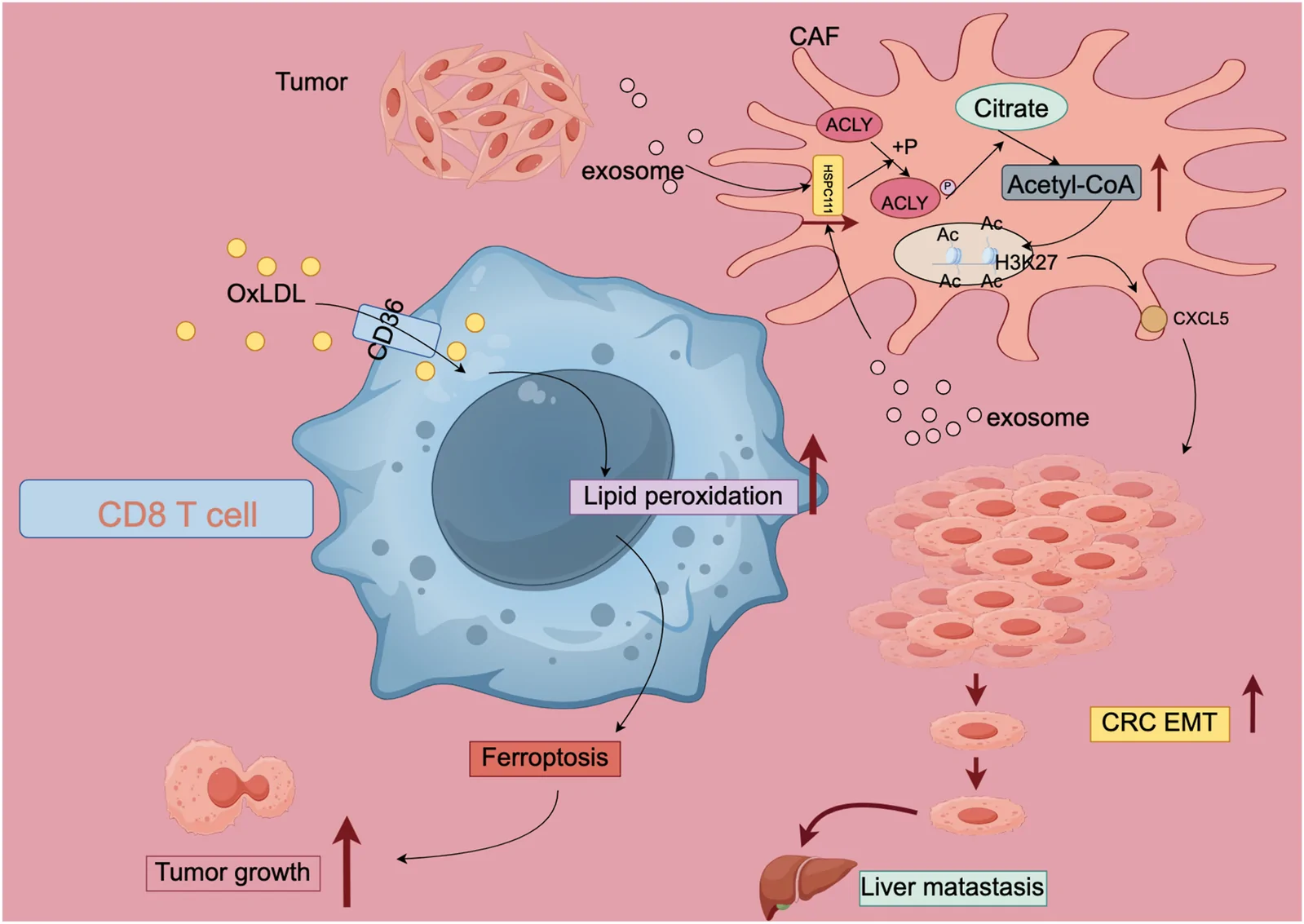
Interaction of lipid metabolism with immune and tumor cells in the tumor microenvironment. Tumor-derived exosomes carrying HSPC111 are taken up by CAFs, where they enhance ACLY phosphorylation, increasing acetyl-CoA. Accumulated acetyl-CoA promotes histone acetylation, which upregulates CXCL5 secretion. These metabolic and epigenetic changes in CAFs facilitate EMT in colorectal cancer and promote liver metastasis. CD8^+^ T cells expressing CD36 uptake OxLDL from the tumor niche, resulting in lipid peroxidation, ferroptosis, and impaired antitumor immunity. ACLY, ATP-citrate lyase; CAFs, cancer-associated fibroblasts; CD36, cluster of differentiation 36; EMT, epithelial–mesenchymal transition; OxLDL, oxidized low-density lipoprotein.

Alterations to lipid metabolism in the immune-edited niche have significant impacts across neoplastic and infiltrating immune populations, particularly cytotoxic lymphocytes. Xu et al demonstrated that CD36^+^ T cells in the tumor microenvironment actively uptake oxidized low-density lipoprotein (OxLDL), leading to lipid peroxidation and triggering ferroptosis. This lipid-induced damage compromises T cell function, but interestingly, this dysfunction can be reversed by antioxidant therapy or by targeting CD36, which has been shown to improve T cell activity *in vitro*.^116^^,^^117^ Furthermore, studies have also revealed that lipid metabolism alterations in CAFs can play a pivotal role in promoting CRC metastasis. The release of exosome HSPC111 (HBV pre-S2 trans-regulated protein 3) from cancer cells influences the metabolic changes in CAFs, thereby accelerating the invasive outgrowth capacity in colorectal carcinoma clones^118^ (Fig. 3). The results underline the importance of lipid reprogramming both in driving neoplastic progression and in orchestrating the tumor microenvironment to facilitate immune evasion and metastatic spread.

#### *Potential clinical applications of lipid metabolic biomarkers*

Alterations in lipid metabolism have been prospectively validated as molecular hallmarks of disease trajectory in CRC. Among the key regulators, sterol regulatory element-binding proteins (SREBPs) have attracted considerable attention as transcriptional biomarkers that orchestrate lipid homeostasis. SREBP1 mainly governs *de novo* fatty acid synthesis, whereas SREBP2 controls cholesterol biosynthesis. Aberrant activation of SREBPs has been linked to malignant progression, chemoresistance, and metastasis. For example, exposure of CRC cells to adipocyte-conditioned medium activates Akt/p70S6K signaling, leading to SREBP1 up-regulation and reduced sensitivity to 5-FU.^119^ Similarly, FUT2-driven fucosylation stabilizes mature SREBP1 via YAP/TAZ signaling, thereby enhancing fatty acid synthesis and promoting proliferation and metastasis.^120^ These results emphasize SREBPs not only as biomarkers reflecting lipid metabolic reprogramming but also as actionable therapeutic targets. Indeed, small-molecule inhibitors such as Fatostatin^121^^,^^122^ and Betulin,^123^ which block SREBP activation and processing, respectively, have demonstrated preclinical efficacy in suppressing lipogenesis and tumor progression.

Beyond transcriptional regulators, several enzymatic regulators previously discussed in the subsections “Role of CPT1/2 in fatty acid oxidation and tumor progression” and “Rewiring of carbon flux and lipid biosynthetic networks in CRC”—notably CPT1/2, ACLY, and FASN—have also emerged as clinically relevant biomarkers in CRC. Rather than reiterating their metabolic functions, it is important to highlight their translational significance: CPT1/2 expression reflects metabolic flexibility under nutrient stress, ACLY levels correlate with tumor aggressiveness and potential epigenetic regulation, and FASN overexpression is strongly associated with poor prognosis. Collectively, these enzymes exemplify how key drivers of lipid metabolism can also serve as diagnostic and prognostic indicators in CRC.

Building on these canonical enzymes, recent studies have identified additional lipid metabolism-related molecules with significant clinical relevance. For instance, adiposity correlates with adverse clinical outcomes in CRC, and dysregulated lipid metabolism may provide a mechanistic link. A 2022 study revealed that microsomal triglyceride transfer protein (MTTP) is significantly up-regulated in plasma-derived exosomes from CRC patients with high adiposity. MTTP overexpression promotes resistance to ferroptosis by increasing the expression of glutathione peroxidase 4 (GPX4) and xCT, thereby reducing lipid peroxidation. Notably, inhibition of MTTP *in vivo* restored chemotherapy sensitivity, highlighting its potential as a biomarker for chemoresistance and a therapeutic target in metabolically reprogrammed CRC^124^ (Fig. 2).

Likewise, peroxisome proliferator-activated receptor gamma (PPARγ), a lipid-sensing nuclear receptor and a key regulator of fatty acid metabolism, has been mechanistically linked to tumorigenic advancement. Aberrant activation of PPARγ signaling contributes to CRC cell proliferation and survival. Recent *in vitro* evidence shows that pharmacological inhibition of PPARγ can suppress malignant phenotypes in CRC cells, suggesting its potential as both a diagnostic marker and a therapeutic target in lipid-driven tumor progression.^125^, ^126^, ^127^ In addition, the protein tyrosine phosphatase receptor type O (PTPRO), a tumor suppressor, has been reported to modulate CRC development by reprogramming lipid metabolism. Specifically, PTPRO silencing activates the AKT/mTOR/SREBP1/ACC1 and MAPK/PPARα/ACOX1 signaling axes, promoting lipogenesis and FAO pathways that facilitate tumor growth and metabolic adaptation^128^ (Fig. 2).

Furthermore, emerging evidence suggests a direct link between cholesterol metabolism and intestinal stem cell behavior. Studies have shown that modulation of cholesterol biosynthesis can promote intestinal stem cell expansion. For instance, a deficiency of phospholipid remodeling enzyme (LPCAT3) leads to enhanced cholesterol synthesis and concomitant increases in stem cell proliferation.^129^ This finding underscores the function of lipid metabolic enzymes not only in tumor metabolism but also in the regulation of cancer-initiating cell populations. Importantly, cholesterol metabolism also contributes to metastatic progression, molecular subtype specificity, and immune regulation in CRC. In liver metastases, hepatocyte growth factor (HGF) from the hepatic microenvironment activates the c-Met/phosphoinositide 3-kinase (PI3K)/protein kinase B (AKT)/mTOR axis, which in turn stimulates SREBP2-dependent cholesterol biosynthesis. Relative to matched primaries, liver lesions exhibit higher SREBP2 and its targets (*e.g.*, HMGCR/HMGCS), increased total/free cholesterol, and up-regulation of LDLR/SRB1, whereas such induction is minimal in brain or lymph-node metastases. Functionally, genetic SREBP2 silencing and pharmacologic blockade of the pathway (Betulin; Simvastatin) reduce liver metastatic burden across orthotopic and intrasplenic models, with cholesterol add-back rescuing growth upon SREBP2 knockdown. These findings establish a liver-specific cholesterol program that is HGF–c-Met/PI3K/AKT/mTORC–SREBP2 dependent.^130^ Moreover, cholesterol metabolism is particularly enriched in APC/KRAS-mutant CRC, where proprotein convertase subtilisin/kexin type 9 (PCSK9) disrupts cholesterol homeostasis, drives the accumulation of geranylgeranyl pyrophosphate, and activates the KRAS/MEK/ERK cascade to enhance tumor growth. Combined inhibition of PCSK9 and cholesterol-lowering agents has demonstrated potential in suppressing tumor progression in this molecular subtype, identifying PCSK9 as a novel therapeutic target.^131^ Extending this cholesterol framework to anti-tumor immunity, Zhou et al demonstrated that dual-specificity phosphatase 18 (DUSP18) sustains cholesterol biosynthesis in CRC. Inhibition of DUSP18 with the FDA-approved agent Lumacaftor synergizes with anti-PD-1 therapy to potentiate tumor immune surveillance *in vivo*.^132^ Overall, these findings illustrate that cholesterol metabolism in CRC extends beyond tumor metabolic reprogramming to encompass metastatic adaptation, subtype-specific vulnerabilities, and immune evasion, offering multiple avenues for therapeutic intervention.

In addition to enzymatic regulators, lipid droplet dynamics are increasingly recognized as important markers of metabolic reprogramming in CRC. Lipid droplets serve as critical organelles for the coordination of lipid synthesis, storage, and signaling. Accumulation of lipid droplets has been detected in CRC cells, particularly in cancer stem cells, where it may support tumor initiation and resistance to treatment. Moreover, loss of the fat-mobilizing protein α/β-hydrolase domain-containing 5 (Abhd5) has been shown to impair mitochondrial β-oxidation, resulting in a metabolic shift toward aerobic glycolysis that enhances neoplastic expansion and dissemination.^133^, ^134^, ^135^, ^136^ Placed in the broader context of lipid metabolism, this collective evidence emphasizes that lipid metabolic rewiring concurrently fuels tumor progression and yields biomarkers with tangible clinical potential. Moving forward, rigorous validation of lipid-based biomarkers in large patient cohorts and prospective clinical trials will be critical to establishing their prognostic and therapeutic value in CRC.

#### *Amino acid metabolic reprogramming: Multifaceted roles in CRC*

Amino acid metabolism is extensively reprogrammed in CRC to sustain neoplastic biomass accrual, maintain redox balance, bypass host immunosurveillance, and adapt to a dynamic tumor microenvironment. Beyond their canonical role as substrates for protein synthesis, specific amino acids, including glutamine, serine, glycine, tryptophan, and asparagine, serve as critical regulators of oncogenic signaling and cellular homeostasis. These pathways intersect with central carbon metabolism and exert downstream effects on epigenetic remodeling, oxidative stress response, and immunoregulatory networks. Growing evidence indicates that such metabolic rewiring contributes to drug resistance and is shaped by both tumor-intrinsic signaling cascades and extrinsic cues such as microbiota-derived metabolites.^137^^,^^138^ In this section, we synthesize current insights by organizing amino acid metabolic reprogramming into six mechanistic axes: glutamine metabolism and its immunometabolic implications; serine–glycine metabolism and nucleotide biosynthesis; tryptophan–aryl hydrocarbon receptor (AHR) signaling and immune evasion; arginine–nitric oxide pathway and epigenetic reprogramming; asparagine metabolism reprogramming and immune modulation; and tumor–microbiota amino acid crosstalk. These thematic clusters highlight the multifaceted roles of amino acid metabolism alongside druggable vulnerabilities in CRC.

#### *Glutamine metabolism and its immunometabolic implications in CRC*

**Figure 4:**
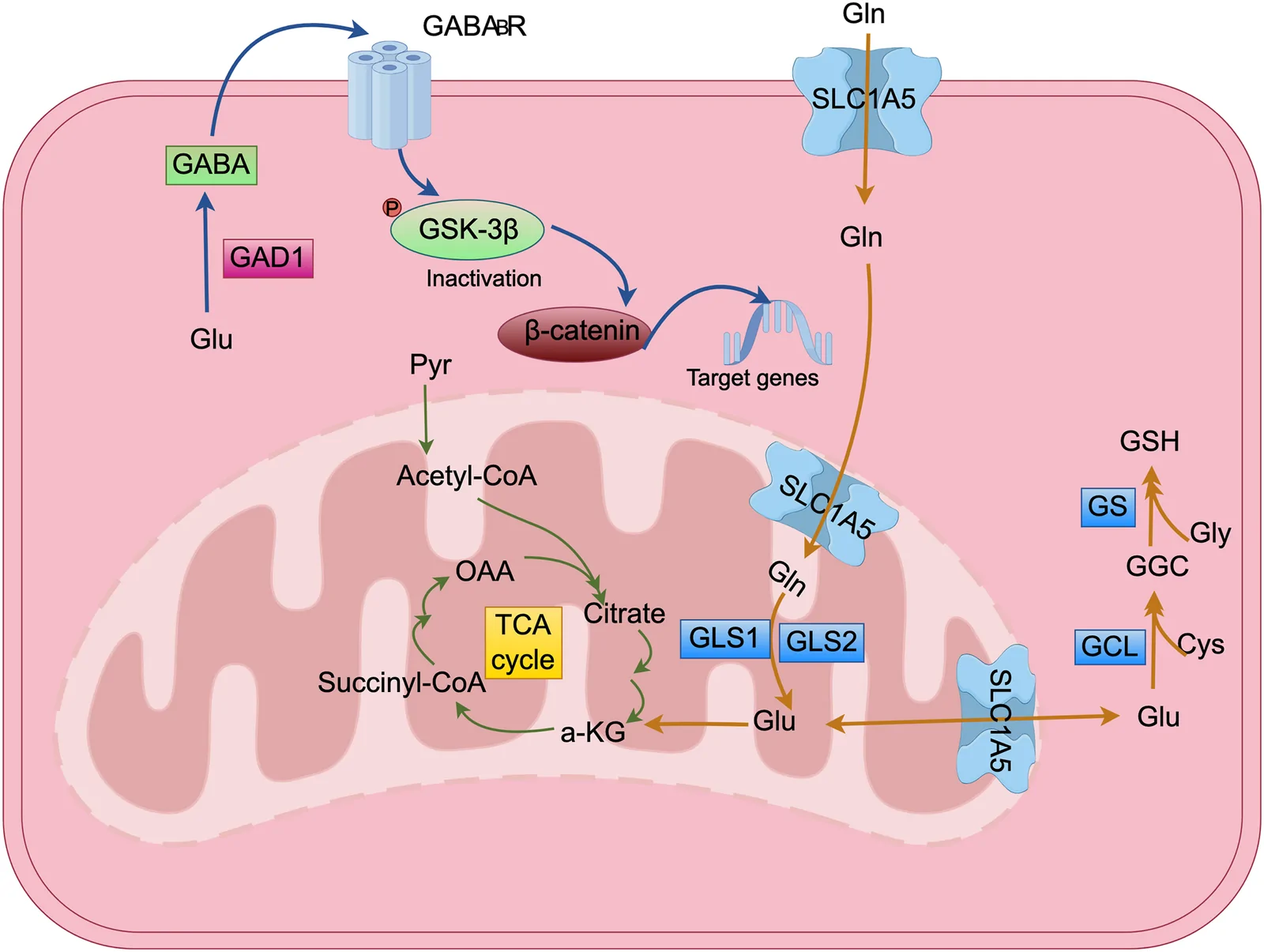
Glutamine metabolism. Glutamine is imported into cells mainly via transporters such as SLC1A5 and converted into glutamate by GLS. Glutamate fuels the TCA cycle to sustain energy production and supports GSH synthesis to maintain redox balance. In addition, the GAD1–GABA axis activates β-catenin signaling, further promoting tumor growth and immune evasion. Pharmacological inhibition of GLS or GAD1 has been shown to sensitize colorectal cancer cells to chemotherapy and immunotherapy, underscoring glutamine metabolism as both a driver of tumor progression and therapeutic target. GLS, glutaminase; GSH, glutathione; GAD1, glutamate decarboxylase 1; GABA, γ-aminobutyric acid; TCA, tricarboxylic acid.

Enhanced glutamine metabolism is a well-recognized metabolic adaptation in cancer, where glutamine operates as a pivotal metabolic substrate second only to glucose in importance. Scientific studies have demonstrated that glutamine metabolism is essential for tumor growth, invasion, autophagy, and immune evasion.^76^ Glutamine enters cells via transporters like SLC1A5 and is converted into glutamate by glutaminase (GLS).^139^ In cancer, p53 induces GLS2 expression,^140^ while c-Myc up-regulates GLS,^141^ both promoting glutamine metabolism and boosting antioxidant capacity via GSH^76^ (Fig. 4). In CRC, the up-regulation of GLS plays a critical role in tumor growth and survival. Consequently, GLS has been proposed as a promising metabolic biomarker, and its inhibition may represent a potential therapeutic strategy by targeting the metabolic vulnerabilities. Recent preclinical research has shown the potential of GLS inhibitors. The combination of GLS inhibitors (such as BPTES) with glucose metabolism inhibitors within a nanoparticle system, as well as GLS1 inhibitors like CB-839, has shown promise in reversing chemoresistance, reprogramming immunosuppressive stromal networks, and synergizing with anti-PD1 therapy in CRC animal models.^142^^,^^143^ Although these results are encouraging, the clinical application of GLS inhibitors in CRC remains in the early development stage. Additional human studies remain imperative to evaluate their efficacy–toxicity profile. In parallel, glutamate-derived γ-aminobutyric acid (GABA) has emerged as another immunometabolic mediator linking glutamine metabolism to oncogenic signaling in CRC. Glutamate decarboxylase 1 (GAD1), which catalyzes the conversion of glutamate into GABA, is aberrantly overexpressed in CRC cells. This GAD1–GABA axis has been shown to activate β-catenin signaling via the GABA receptor (Fig. 4), promoting tumor proliferation and facilitating immune escape by impairing CD8^+^ T cell infiltration. *In vivo* studies have demonstrated that targeting GAD1 or GABA receptor signaling can significantly enhance the anti-tumor efficacy of immunotherapies such as anti-PD-1 antibodies, indicating a potential strategy to overcome immune resistance in CRC.^144^ Collectively, these findings highlight the dual role of glutamine metabolism in sustaining tumor bioenergetics and shaping the immunosuppressive tumor microenvironment. Targeting key metabolic enzymes such as GLS and GAD1 presents an exciting avenue for future therapeutic interventions.

#### *Serine–glycine metabolism and nucleotide biosynthesis in CRC*

Studies indicate that serine is a crucial building block for protein and nucleotide production and actively participates in regulating glycolysis. The binding of serine to PKM2 enhances its activity, thereby accelerating the glycolytic process. Under conditions of serine deficiency, PKM2 activity decreases, prompting cells to shift their metabolic mode by reducing glycolysis and increasing OXPHOS to maintain energy balance and support continued growth.^26^ Glycine, serine, and other single-carbon donors derived from the folate pathway are strongly linked to metastasis and unfavorable outcomes in malignancies such as CRC.^145^, ^146^, ^147^ Additionally, the *de novo* production of serine and downstream single-carbon metabolism are critical for purine depletion-induced cell migration.^148^ Tumor cells frequently boost the metabolism of serine and glycine for nucleotide production and redox equilibrium.^149^ This tight coupling between serine–glycine metabolism and cellular redox balance highlights a role for tumor suppressors, such as p53 and p73, in orchestrating metabolic adaptation under nutrient stress. Under serine starvation, p53 activates the p53–p21 axis to induce cell-cycle arrest, thereby diverting limited serine stores toward glutathione synthesis and maintaining redox balance. In parallel, its family member p73 enhances *de novo* serine biosynthesis, supporting nucleotide production and cell proliferation under metabolic stress. These findings emphasize that serine–glycine metabolism is not only a biosynthetic hub but also a critical node of stress adaptation, tightly integrated with tumor suppressor signaling. Consequently, tumors harboring defects in p53 or p73 may exhibit selective vulnerabilities to serine depletion strategies, providing a rationale for exploring dietary or pharmacological interventions in CRC.^150^ In CRC, key enzymes in the serine–glycine pathway, such as PHGDH and phosphoserine aminotransferase 1 (PSAT1), are frequently overexpressed and correlated with tumor progression. Inhibiting PHGDH has been shown to enhance the sensitivity of CRC cells to radiotherapy.^151^ Alcohol dehydrogenase 1C (ADH1C) has been identified as a potential tumor suppressor in CRC, exerting its anti-cancer effects by inhibiting PHGDH and PSAT1. *In vitro* and *in vivo* studies have demonstrated that overexpression of ADH1C holds promise as a therapeutic strategy for CRC.^152^ However, despite the promising potential of these biomarkers, clinical trials to evaluate their therapeutic implications in CRC are still lacking.

#### *Tryptophan–AHR signaling and immune evasion in CRC*

**Figure 5:**
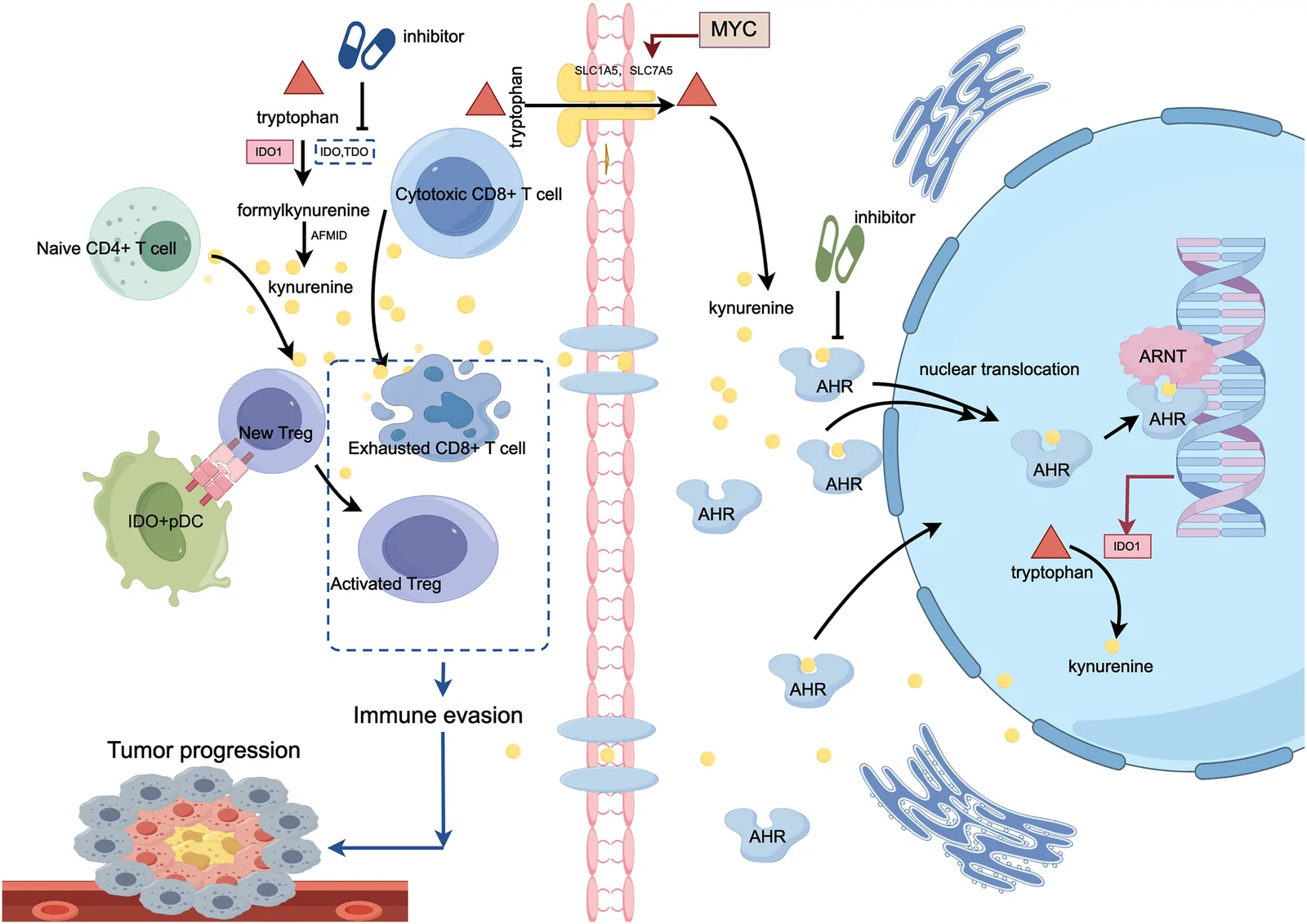
Trp–Kyn–AHR signaling drives immune evasion in colorectal cancer. In colorectal cancer, IDO/TDO catalyze tryptophan to kynurenine, while MYC upregulates transporters (SLC7A5/SLC1A5) and AFMID to enhance intracellular Kyn accumulation. Kyn activates AHR, inducing its nuclear translocation with ARNT. Elevated Kyn also stimulates Treg activation and CD8^+^ T cell exhaustion, facilitating immune evasion. Representative inhibitors targeting this pathway include CH223191 and BAY 2416964 (AHR inhibitors), as well as b-AP15 (USP14 inhibitor), which restore antitumor immunity in preclinical models. IDO, indoleamine 2,3-dioxygenase; TDO, tryptophan 2,3-dioxygenase; AFMID, arylformamidase; AHR, aryl hydrocarbon receptor; ARNT, aryl hydrocarbon receptor nuclear translocator; Treg, regulatory T cell; USP14, ubiquitin-specific protease 14.

Numerous research findings have demonstrated that alterations in the amino acid pathway impact cancerous and immune cells within the tumor niche, contributing to immune evasion.^153^ For instance, indoleamine 2,3-dioxygenase (IDO) and tryptophan 2,3-dioxygenase (TDO) catalyze the conversion of tryptophan into kynurenine, with AHR expressed in CRC (Fig. 5). Niranjan Venkateswaran et al discovered that MYC elevates intracellular concentrations of tryptophan and its kynurenine pathway derivatives by up-regulating tryptophan transporters SLC7A5, SLC1A5, and arylformamidase (AFMID). These proteins, along with kynurenine, are significantly elevated in malignant CRC cells in comparison with adjacent normal tissues in patients. Studies have shown that kynurenine facilitates the nuclear translocation of AHR in CRC, and inhibiting AHR-kynurenine interaction using CH223191 decreases colon cancer cell expansion. Furthermore, kynurenine’s immunosuppressive effects in the tumor microenvironment are largely AHR-mediated. BAY 2416964 inhibits AHR activation, restoring immune cell function and showing anti-tumor effects in preclinical models^153^, ^154^, ^155^, ^156^ (Fig. 5). Currently, BAY 2416964 is being evaluated in a phase I clinical trial (NCT04999202) in combination with the immune checkpoint inhibitor pembrolizumab for the treatment of advanced solid tumors. However, no AHR inhibitors have yet progressed to phase II trials specifically targeting CRC, and their clinical utility in CRC remains to be established. Additionally, the overexpression of proteasome-associated deubiquitinating enzyme USP14 stabilizes IDO1 protein, which not only promotes tryptophan metabolism in CRC but also hinders the activity of cytotoxic T cells, thereby facilitating immune evasion.^157^ Current preclinical studies, both *in vitro* and *in vivo*, have demonstrated that the USP14 inhibitor b-AP15 can reduce the malignancy of CRC.^158^ However, future investigations should focus on the development of safer and more selective USP14 inhibitors, along with thorough evaluation of their clinical efficacy and safety.

#### *Arginine–nitric oxide pathway and epigenetic reprogramming in CRC*

Many studies have indicated that the l-arginine pathway may aid in the initiation and progression of CRC.^159^, ^160^, ^161^, ^162^ Małgorzata Krzystek-Korpacka and colleagues further discovered metabolic reprogramming in the l-arginine/nitric oxide (NO) pathway in both CRC and tumor-adjacent tissues, marked by the overexpression of ARG1, NOS2, dimethylarginine hydrolases (DDAH1 and DDAH2), and highlighted the involvement of protein arginine methyltransferases, specifically PRMT1 and PRMT5, in this process. Furthermore, they showed that these alterations could be regulated by novel oxicam-based nonsteroidal anti-inflammatory drug derivatives.^162^ These findings not only highlight the molecular reprogramming of amino acid metabolism in CRC but also point to novel therapeutic strategies. The use of nonsteroidal anti-inflammatory drug-based thiazole derivatives as a therapeutic approach to regulate the l-arginine/NO pathway offers a promising avenue for CRC treatment, potentially leading to more targeted therapies that address specific metabolic shifts in CRC cells.

#### *Asparagine metabolism reprogramming and immune modulation in CRC*

In recent years, reprogramming of asparagine metabolism has emerged as a pivotal mechanism influencing immune regulation in CRC. Zhou et al demonstrated that asparagine promotes immune evasion in CRC by activating the non-receptor tyrosine kinase SRC, which subsequently triggers the ERK/ETS2 signaling cascade. This signaling pathway facilitates the expansion of myeloid-derived suppressor cells, thereby suppressing CD8^+^ T cell function and fostering an immunosuppressive tumor microenvironment.^163^ Conversely, deprivation of asparagine has been shown to enhance the metabolic adaptability and anti-tumor efficacy of CD8^+^ T cells. Mechanistically, this effect involves activation of the NRF2 signaling pathway, reprogramming of glucose and glutamine metabolism, and up-regulation of nucleotide biosynthesis—collectively supporting T cell proliferation and effector function.^164^ Insights from single-cell transcriptomic analyses have further revealed that the expression of asparagine synthetase (ASNS) is dynamically regulated during CD8^+^ T cell differentiation. Elevated ASNS expression correlates with the acquisition of effector phenotypes, implicating it as a potential metabolic biomarker predictive of immune responsiveness.^165^ In addition, a preclinical study employing patient-derived xenograft models of APC-mutant CRC reported that combined administration of l-asparaginase and the glycogen synthase kinase-3α (GSK3α) inhibitor BRD0705 led to substantial tumor growth inhibition and prolonged progression-free survival, underscoring the therapeutic promise of targeting asparagine metabolism in CRC.^166^ Although systematic evaluation of l-asparaginase in combination with immune checkpoint inhibitors remains lacking in CRC-specific models, encouraging results from other solid tumors, such as nasopharyngeal carcinoma, provide a compelling rationale for translational exploration in CRC.^167^ Taken together, these findings position asparagine metabolism not only as a key regulator of tumor immune evasion but also as a tractable target for combination immunotherapy strategies. Further investigation into ASNS expression and asparagine-depleting therapies may yield valuable biomarkers and inform novel metabolic-immunotherapeutic approaches for CRC.

#### *Microbiome–amino acid crosstalk and epigenetic regulation in CRC*

In the progression of CRC, metabolic alterations are not limited to amino acid metabolism but are also closely associated with changes in DNA methylation patterns. Previous studies have reported genome-wide hypomethylation in CRC.^168^, ^169^, ^170^ Recently, Liu et al also found that CRC samples showed notably reduced global methylation levels in comparison with adjacent normal tissues. Furthermore, they observed an increase in the l-methionine biosynthesis III pathway.^170^ These findings highlight how changes in methylation patterns and amino acid metabolism are interlinked in CRC progression. Apart from the above findings, Liu et al highlighted a strong correlation between the metabolism of the fecal microbiota and the metabolism of malignant cells in CRC, with a substantial rise in the l-glutamate degradation VIII pathway in cancer cells.^170^ This finding aligns with the research by Sarah Obuya et al, indicating that CRC patients have distinct microbiome and metabolic characteristics compared with healthy controls.^171^ Together, these findings suggest that host–microbiome metabolic crosstalk may contribute to tumor progression via shaping amino acid availability, redox balance, and epigenetic regulation. Therapeutic strategies targeting microbial metabolism or restoring microbial homeostasis might thus represent novel approaches for CRC management.

#### *Adenosine, m*^*6*^*A, and immune modulation in CRC*

In addition to traditional metabolites such as glucose, lipids, and amino acids, adenosine has emerged as a critical metabolic mediator in tumor reprogramming. Extracellular adenosine exerts immunosuppressive effects through its four receptors (A1R, A2AR, A2BR, and A3R), among which A2AR plays a central role in suppressing T cell and NK cell activity, thereby promoting immune evasion in CRC.^172^^,^^173^ Research has indicated that the adenosine/CD73/CD39/adenosine receptor (ARs) axis significantly contributes to immune evasion in CRC. Notably, gene silencing or blockade of CD73 or A2AR has been shown to enhance anti-tumor immunity, inhibit tumor cell proliferation, and improve therapeutic responses.^174^ Hypoxia, a common characteristic in the tumor microenvironment, further amplifies adenosine accumulation by activating hypoxia-inducible factor alpha (HIF-α) and NF-κB signaling, establishing a positive feedback loop that facilitates tumor progression and immune suppression.^175^ Consequently, adenosine metabolism not only serves as a critical component of neoplastic metabolic plasticity but also embodies a fundamental mechanism of immune evasion.

Beyond direct regulation by adenosine metabolism, N6-methyladenosine (m^6^A), a modification at the sixth nitrogen of adenosine in RNA, acts as a pivotal orchestrator of tumor cell metabolism and immune escape. m^6^A modification influences RNA stability, splicing, and translation, which in turn governs neoplastic metabolic adaptation and immune evasion.^176^ Cutting-edge findings establish m^6^A’s profound association with programmed cell death and modulate tumor cell responses to immunotherapy.^177^^,^^178^ Additionally, m^6^A regulators, such as methyltransferase 16 (METTL16), have been implicated in the progression of CRC.^179^ Specifically, the m^6^A reader insulin-like growth factor-II mRNA binding protein 2 (IMP2) stabilizes the zinc finger antisense 1 (ZFAS1)/Obg-like ATPase-1 (OLA1) axis, which is critical for activating the Warburg effect in CRC, thus contributing to their metabolic reprogramming and tumor progression.^180^ In some cases, m^6^A biomarkers have been identified as potential non-invasive biomarkers in cancers, including CRC.^181^^,^^182^ In conclusion, both adenosine metabolism and m^6^A modification are pivotal in tumor metabolic reprogramming. These processes not only influence energy metabolism, such as glycolysis and ATP hydrolysis, but also contribute to immune evasion, enhancing tumor growth and therapeutic resistance. In consequence, combined detection of m^6^A and the CD73/CD39/ARs axis regulators holds significant promise for the diagnosis and therapeutic monitoring of CRC.

### Interaction between signaling pathways and metabolism

#### *The significance of the Wnt signaling pathway in the metabolic reprogramming of CRC*

**Figure 6:**
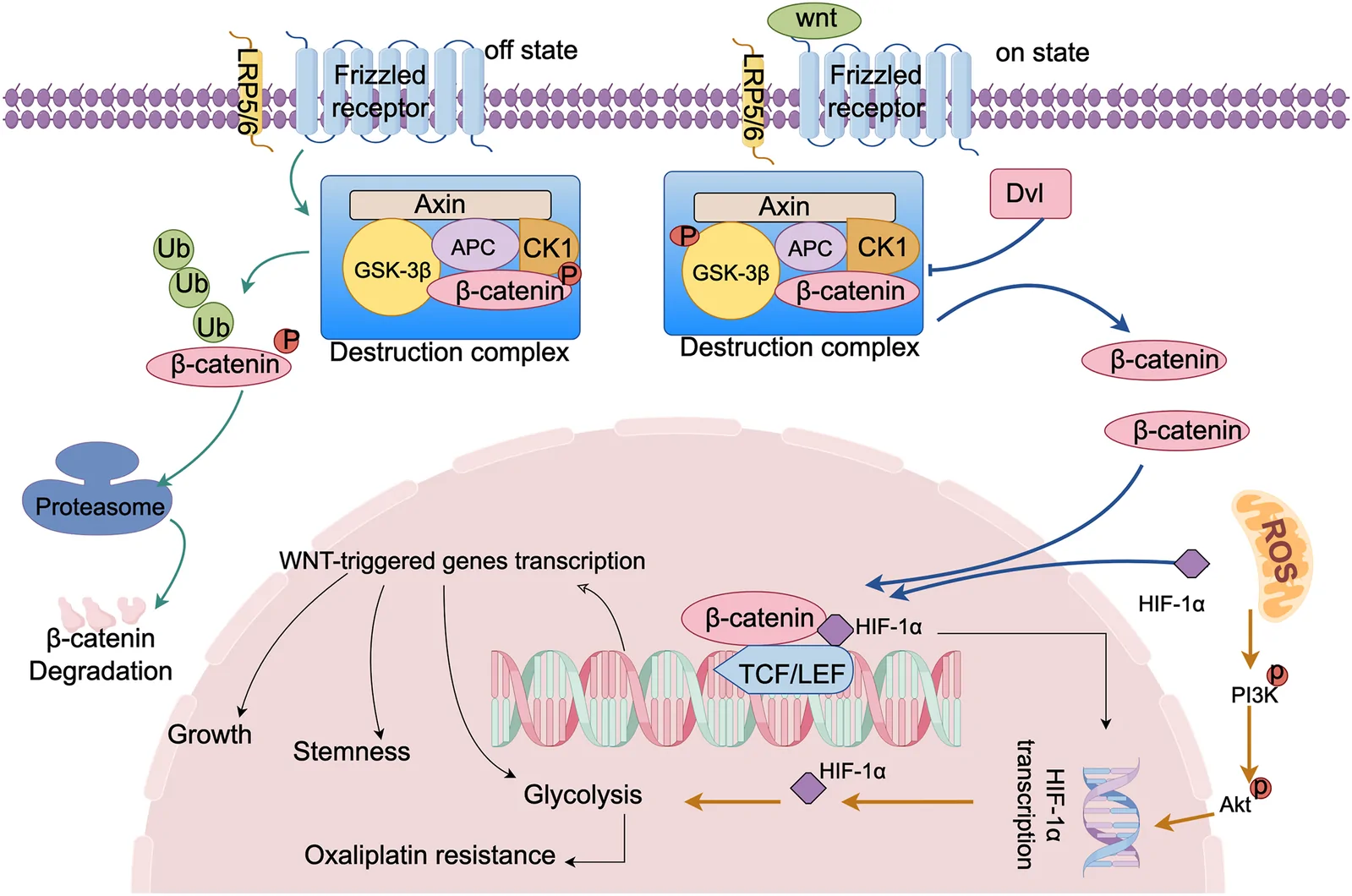
Wnt/β-catenin signaling pathway off and on states. In the absence of Wnt ligands (off state), β-catenin is phosphorylated by the destruction complex (comprising APC, Axin, GSK-3β, and CK1), ubiquitinated, and subsequently degraded by the proteasome. Upon Wnt activation (on state), binding of Wnt to Frizzled and LRP5/6 receptors recruits Dvl, leading to disruption of the destruction complex and stabilization of β-catenin. Stabilized β-catenin translocates into the nucleus, where it interacts with TCF/LEF transcription factors to induce Wnt target genes that regulate cell growth, stemness, glycolysis, and oxaliplatin resistance. Importantly, β-catenin also regulates HIF-1α, and their nuclear cooperation enhances HIF-1α transcriptional activity, further amplifying glycolytic gene expression and metabolic reprogramming. In parallel, ROS and PI3K/Akt signaling promote HIF-1α stabilization, reinforcing the crosstalk between Wnt/β-catenin signaling, hypoxia adaptation, and therapy resistance in colorectal cancer. APC, adenomatous polyposis coli; CK1, casein kinase 1; GSK-3β, glycogen synthase kinase 3β; LRP5/6, low-density lipoprotein receptor-related protein 5/6; Dvl, dishevelled; ROS, reactive oxygen species; HIF-1α, hypoxia-inducible factor 1α; TCF/LEF, T cell factor/lymphoid enhancer-binding factor.

The Wnt pathway is a cellular signaling cascade triggered by proteins with lipid modifications that are secreted by Wnt family members. At its core, this pathway comprises a Wnt protein secreted by the signaling cell, specific receptors present on the target cell membrane, and intracellular signal transducers. Once the ligand is recognized, the signal is transmitted within the cell, resulting in pathway activation and eliciting cellular responses, including mitotic activity, differentiation of cell types, and the regulation of cellular polarity.^183^ Wnt-mediated signaling has a history of over 40 years since its discovery, and our understanding of its mechanisms has significantly deepened.^184^ It is regarded as a crucial regulator of growth, essential for tissue development in animals, and a key driver of most tissue stem cells in mammals. However, alterations in the Wnt signaling cascade are linked to the development of various malignancies, particularly colorectal tumors.^185^ Notably, the APC gene, a crucial neoplastic suppressor gene often mutated in CRC, regulates the Wnt pathway downstream^186^ (Fig. 6). Furthermore, a study conducted in 2012 found that 93% of CRC patients exhibited aberrations in the Wnt pathway.^187^

In the tumor microenvironment, Wnt signaling not merely drives CRC cell growth but simultaneously facilitates metabolic plasticity, empowering malignant progression in hypoxic and nutrient-scarce niches. Studies have shown that Wnt signaling directly regulates glycolysis by activating 3-phosphoinositide-dependent kinase-1 (PDK1), which contributes to the metabolic reprogramming. Specifically, Wnt signaling up-regulates the expression of PDK1, inhibiting the activity of PDH, thereby limiting the conversion of pyruvate to acetyl-CoA. This disruption of the OXPHOS process forces tumor cells to resort to the Warburg pathway to meet bioenergetic demands. As an important target gene of Wnt signaling, PDK1 plays a key role not only in metabolic reprogramming but also in modulating the tumor microenvironment, such as promoting tumor angiogenesis. Studies have found that inhibition of Wnt signaling reduces PDK1 levels, which in turn suppresses the glycolytic phenotype and significantly decreases tumor vessel density. Furthermore, although previous research suggests a correlation between c-Myc up-regulation and PDK1 expression, experimental data indicate that Wnt signaling regulates PDK1 independently of c-Myc, indicating that PDK1 up-regulation is primarily driven by direct Wnt signaling. To mechanistically substantiate the metabolic role of PDK1 under Wnt signaling regulation, the PDK inhibitor dichloroacetate was used. The results showed that dichloroacetate treatment induced metabolic changes similar to those observed with dnLEF/TCF expression, including a marked reduction in glycolytic activity and an increase in OXPHOS, restoring cellular energy balance. Moreover, the combination of Wnt inhibition and dichloroacetate treatment sensitized CRC cells to chemotherapeutic challenge, further supporting the close link between Wnt signaling and metabolic plasticity.^188^^,^^189^

The HIF-1α signaling pathway is a classic response to hypoxic conditions. HIF-1α is regulated by β-catenin, and the collaboration of nuclear β-catenin and HIF-1α enhances the latter’s transcriptional activity (Fig. 6). Research by Dong et al suggests that the alteration of cellular metabolism promoting resistance to fluorouracil in CRC occurs through a non-classical up-regulation of HIF-1α, involving reactive oxygen species (ROS) and the Wnt/β-catenin regulatory network, independent of extracellular oxygen availability.^36^ Similarly, Li et al found that CPT2 down-regulation promoted CRC resistance to oxaliplatin by promoting glycolysis through a ROS/Wnt/β-catenin-driven metabolic shift.^60^

In the pathogenesis and progression of CRC, the Wnt signaling pathway is not only modulated by intrinsic cellular factors but also influenced by microbial-derived metabolites. Accumulating evidence indicates that microbial metabolites such as SCFAs, secondary bile acids, and tryptophan derivatives can regulate the Wnt/β-catenin pathway through distinct mechanisms, thereby affecting tumor cell proliferation and intestinal barrier integrity. For instance, *Clostridium butyricum*, a butyrate-producing probiotic, generates butyrate that facilitates autophagy-mediated degradation of β-catenin, resulting in the inhibition of Wnt signaling activation and suppression of tumor cell proliferation. This highlights the potential of probiotic-derived metabolites in maintaining intestinal homeostasis and exerting anti-tumor effects.^190^ In contrast, deoxycholic acid, a secondary bile acid produced by bacterial transformation of primary bile acids, has been shown to enhance Wnt/β-catenin signaling, thereby promoting CRC development and progression.^191^ These findings suggest that certain microbial metabolites may play a pro-oncogenic role. Tryptophan metabolites also exert multifaceted effects on Wnt pathway regulation. Indole-3-aldehyde (I3A), derived from microbial metabolism, can activate the AhR/Wnt axis to promote intestinal epithelial proliferation. This effect appears beneficial in mitigating radiation-induced epithelial damage but may concurrently increase the risk of neoplastic transformation under chronic exposure.^192^ Collectively, these studies underscore the significant role of microbial metabolites in modulating the Wnt signaling pathway. These findings offer new perspectives on CRC pathophysiology and support the development of microbiota-targeted strategies modulating Wnt signaling.

#### *The significance of PI3K/Akt signaling cascade in metabolic reprogramming of CRC*

**Figure 7:**
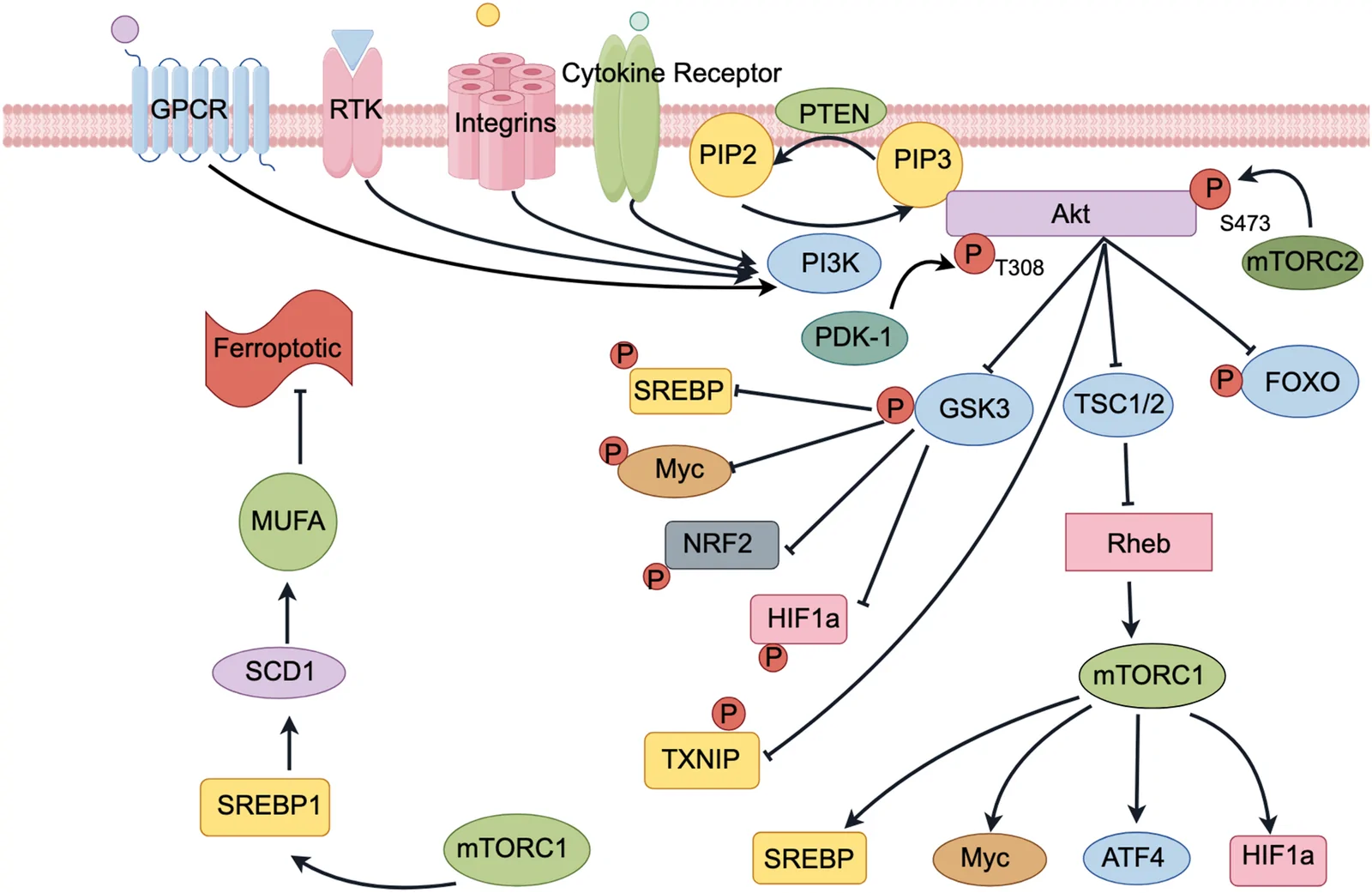
PI3K/Akt signaling pathway. The PI3K/Akt pathway integrates extracellular signals from RTKs, GPCRs, integrins, and cytokine receptors to regulate cellular metabolism and survival. Upon activation, PI3K converts PIP2 into PIP3, which recruits Akt to the plasma membrane. Akt is phosphorylated at T308 by PDK1 and at S473 by mTORC2, achieving full activation. Activated Akt phosphorylates multiple downstream effectors, including GSK3, TSC1/2, and FOXO transcription factors, thereby promoting mTORC1 activity, inhibiting catabolic signaling, and enhancing anabolic metabolism. mTORC1 subsequently activates key transcriptional regulators such as SREBP, Myc, ATF4, and HIF-1α, which drive glycolysis, lipid biosynthesis, and redox homeostasis. In parallel, Akt suppresses TXNIP to enhance glucose uptake. Notably, mTORC1–SREBP1 signaling promotes SCD1-mediated MUFA synthesis, which confers resistance to ferroptosis. Collectively, this signaling cascade illustrates how PI3K/Akt integrates growth signals with metabolic reprogramming and therapy resistance in colorectal cancer. RTK, receptor tyrosine kinase; GPCR, G protein-coupled receptor; PIP2, phosphatidylinositol 4,5-bisphosphate; PIP3, phosphatidylinositol 3,4,5-trisphosphate; PI3K, phosphoinositide 3-kinase; PTEN, phosphatase and tensin homolog; PDK1, 3-phosphoinositide-dependent kinase-1; mTORC1/2, mechanistic target of rapamycin complex 1/2; FOXO, forkhead box O; SREBP, sterol regulatory element-binding protein; TXNIP, thioredoxin-interacting protein; HIF-1α, hypoxia-inducible factor 1α; MUFA, monounsaturated fatty acid; SCD1, stearoyl-CoA desaturase 1.

The PI3K/Akt cascade represents the predominant hyperactivated signaling network across human malignancies. In normal physiological contexts, it responds to biological signals such as insulin, orchestrating pivotal biochemical processes like glucose processing, macromolecular synthesis, and oxidative homeostasis. In neoplastic cells, oncogenic activation of this pathway alters the metabolic landscape by increasing the activity of carriers and enzymes, which meet the biosynthetic and energetic requirements of proliferating cells. This signaling network, initiated through receptor tyrosine kinases (RTKs), receptors for cytokines, integrins, and G protein-coupled receptors (GPCRs), is crucial for cell survival and growth.^193^, ^194^, ^195^ PI3K activation at the plasma membrane phosphorylates PIP2 to generate PIP3, while PTEN counteracts this signaling by dephosphorylating PIP3 back to PIP2. Akt, a central effector in this cascade, binds PIP3 and is phosphorylated at T308 by PDK1 and at S473 by mTORC2^194^^,^^196^, ^197^, ^198^ (Fig. 7).

Once activated, Akt regulates metabolism both by directly phosphorylating metabolic enzymes and by indirectly modulating transcription factors such as mTORC1, GSK3, and Forkhead box O (FOXO).^199^ Specifically, Akt phosphorylates TSC complex subunit 2 (TSC2), disrupting its interaction with Ras homolog enriched in brain (RHEB), resulting in increased RHEB-GTP levels that activate mTORC1, a critical modulator of anabolic metabolism and cellular expansion.^200^^,^^201^ Additionally, GSK3, the first identified Akt substrate, is inhibited by Akt phosphorylation, preventing it from degrading downstream targets.^202^, ^203^, ^204^ Akt also phosphorylates FOXO1, FOXO3A, and FOXO4, sequestering them from the nucleus and inhibiting their gene expression^205^^,^^206^ (Fig. 7).

Akt facilitates glucose uptake through glucose transporter type 1 (GLUT1) and GLUT4. GLUT1 is widely expressed and often up-regulated in cancer,^207^ while GLUT4 primarily aids in insulin-mediated glucose clearance in muscle and adipose tissues.^194^^,^^208^ Recent studies identify thioredoxin-interacting protein (TXNIP) as a direct target of Akt that regulates the trafficking of GLUT1 and GLUT4. By promoting the internalization of GLUT1, TXNIP inhibits glucose uptake. Akt-mediated phosphorylation of TXNIP results in a decrease in TXNIP activity, raising the surface expression of GLUT1 and GLUT4, which enhances glucose uptake in various cell types.^209^, ^210^, ^211^ Besides facilitating glucose import, Akt orchestrates vital glycolytic processes through the phosphorylation of specific catalysts. Hexokinase 2 (HK2) is up-regulated in various human cancers, including CRC, where it is essential for tumor growth and metastasis. Akt promotes HK2 association with mitochondrial voltage-dependent anion channels, ensuring local ATP availability for glucose phosphorylation and maintaining mitochondrial integrity.^212^^,^^213^ Furthermore, phosphofructokinase-1 (PFK1) is the first key step in glycolysis, and Akt indirectly enhances its activity by phosphorylating PFKFB2 or PFKFB3, thereby promoting high glycolytic flux.^214^, ^215^, ^216^ Together, these nodes position PI3K/Akt as a proximal controller of glycolytic flux and mitochondrial–glycolytic coupling in CRC.

Through its broad regulation of cellular metabolism, the PI3K/Akt signaling cascade not only drives metabolic reprogramming but also shapes redox homeostasis and ferroptosis sensitivity. ROS serve as key controllers of various cellular mechanisms, and their heightened production is a hallmark of many pathological processes. ROS are integral to oncogenesis and malignancy advancement, with increased production potentially leading to mitochondrial damage and dysfunction.^36^^,^^217^^,^^218^ To counteract excess ROS, NADPH synthesized through the oxidative pentose phosphate pathway (PPP) helps maintain intracellular ROS balance and protect mitochondrial function.^36^^,^^219^^,^^220^ Moreover, the PI3K/Akt cascade is central to regulating substance and energy metabolism,^194^ with the up-regulation of HIF-1α and enhanced glycolytic flux relying on its activation. In research by Dong et al, it was suggested that 5-FU-resistant cells exist in a state of elevated oxidative stress, evidenced by noticeable mitochondrial dysfunction and an up-regulation of PPP. Further investigations revealed that the build-up of ROS in 5-FU-resistant cells is partly due to significant reductions in the expression and activity of mitochondrial ROS-scavenging enzymes. Importantly, their findings indicate a close relationship between ROS and the dysregulation of the PI3K/Akt cascade in 5-FU-resistant colorectal adenocarcinoma lineages^36^^,^^221^ (Fig. 7). Ferroptosis induction has been validated as a therapeutic paradigm for CRC. Inhibiting PI3K, AKT, or mTOR enhances sensitivity to ferroptosis by reducing the synthesis of MUFAs that typically inhibit this process. Furthermore, various small molecules have shown effectiveness in targeting CRC by down-regulating the PI3K–AKT–mTOR pathway^186^^,^^222^ (Fig. 7). These findings suggest that the PI3K/Akt cascade acts as a central hub linking redox homeostasis with metabolic signaling networks. The following section will discuss how PI3K/Akt interacts with key regulators such as AMPK and the Wnt/β-catenin pathway to coordinate energy metabolism, cell proliferation, and tumor progression in CRC.

The PI3K/Akt axis functions as a critical nexus for integrating microenvironmental mitogenic cues into tumor cell metabolic reprogramming in CRC. Accumulating experimental data suggests that PI3K/Akt signaling not only drives anabolic processes such as protein and lipid synthesis but also actively interacts with other metabolic regulators to promote tumor progression. One of the key regulatory interactions occurs between PI3K/Akt and AMPK, a central energy sensor activated under metabolic stress. AMPK exerts tumor-suppressive functions by promoting catabolic metabolism and inhibiting glycolysis and biosynthesis. Upon activation, AMPK suppresses the PI3K/Akt/mTOR axis, thereby antagonizing anabolic signaling and restricting CRC cell proliferation.^223^ Pharmacologic agents like metformin and DT-13 exert anti-CRC effects partly by activating AMPK and attenuating PI3K/Akt/mTOR signaling.^224^^,^^225^ Conversely, loss of upstream regulators such as Abhd5 leads to AMPK inactivation and enhanced glycolytic flux, reinforcing the reciprocal regulation between these two pathways. Recent findings further expand this regulatory network by demonstrating that PI3K/Akt signaling also modulates the Wnt/β-catenin pathway. Exposure to the environmental carcinogen MC-LR has been shown to activate PI3K/Akt, leading to phosphorylation-mediated inhibition of GSK3β, stabilization of β-catenin, and subsequent up-regulation of downstream effectors c-Myc and cyclin D1. Notably, pharmacological inhibition of PI3K suppressed both Akt and β-catenin signaling, suggesting that PI3K/Akt acts upstream of Wnt/β-catenin in CRC cells.^226^ These findings highlight PI3K/Akt signaling as a central node that coordinates with AMPK and Wnt/β-catenin pathways to regulate energy metabolism, cell proliferation, and tumor progression. Understanding this integrated signaling network establishes the rationale for designing combination therapies targeting metabolic vulnerabilities in CRC.

Given its pivotal involvement in bioenergetic remodeling and malignant advancement, the PI3K/Akt pathway has risen to prominence as a promising therapeutic target in CRC. Numerous clinical trials are currently evaluating PI3K inhibitors as potential treatments for CRC. For example, the PI3K inhibitor alpelisib has elicited durable tumor responses in a Phase I clinical trial involving patients with advanced CRC harboring PIK3CA mutations.^227^ This trial highlights the therapeutic potential of targeting the PI3K pathway in CRC, particularly for patients with specific genetic alterations. However, given the complexity of the PI3K/Akt signaling network, targeting its upstream or downstream regulators, such as AMPK activators, may also offer promising therapeutic strategies. The involvement of PI3K signaling in regulating metabolic reprogramming provides a strong theoretical foundation for the clinical translation of PI3K inhibitors, as well as agents targeting related pathways like AMPK, in CRC treatment.

#### *NF-κB pathway as a driver of metabolic alterations in CRC*

The NF-κB signaling system is composed of two main pathways: the classical and non-classical. In the classical signaling cascade, phosphorylation of IκBα triggers its degradation, allowing the NF-κB heterodimer—typically composed of RelA (p65) and p50—to translocate into the nucleus and regulate gene transcription. In the non-classical pathway, phosphorylation of IKKα leads to the conversion of p100 to p52, which then activates the p52–RelB complex to translocate into the nucleus.^228^^,^^229^ In addition to regulating immune responses and inflammation, NF-κB activation has been found to be aberrantly triggered in several types of human malignancies recently.^230^ Evidence suggests that activation of this pathway promotes the emergence of CRC.^231^^,^^232^ Accumulating evidence reveals that the coupling of the NF-kB pathway in CRC concurrently drives profound metabolic alterations. A representative example is the retinoic acid-inducible gene I (RIG-I), which is aberrantly overexpressed in CRC. Loss-of-function studies demonstrated that depletion of RIG-I significantly suppresses glycolytic activity, as indicated by reduced extracellular acidification rate, lactate production, and ATP levels in CRC cell lines (CACO2 and SW1116). Mechanistically, RIG-I knockdown down-regulated the expression of glycolysis-associated proteins, including HIF-1α, PKM2, GLUT1, and LDHA, while simultaneously blocking NF-κB p65 nuclear translocation. Conversely, enforced RIG-I overexpression in SW480 cells markedly enhanced glycolytic flux, increased lactate and ATP production, and up-regulated the same glycolytic regulators. Importantly, pharmacological inhibition of NF-κB with PDTC abrogated these effects, confirming that RIG-I exerts its metabolic and pro-proliferative functions in CRC at least partly through NF-κB signaling. Collectively, these findings highlight a direct mechanistic link between RIG-I-mediated NF-κB activation and enhanced glycolytic reprogramming in CRC, providing a rationale for targeting this axis to impair tumor metabolic fitness and progression.^233^ Recent work has further clarified the metabolic role of metadherin (MTDH) in CRC. Instead of functioning solely as an oncogenic driver, MTDH also enhances glycolytic capacity, as reflected by increased glucose utilization and lactate output in CRC cells. Importantly, this effect is strongly dependent on NF-κB p65: once p65 was silenced, the glycolytic activation mediated by MTDH was largely abolished. These findings suggest that MTDH does not act in isolation but rather rewires cellular metabolism through an MTDH–NF-κB axis. By reinforcing glycolytic flux, this pathway provides tumor cells with metabolic adaptability, supporting both their growth and stemness properties. From a translational perspective, disrupting this axis may represent an attractive strategy to suppress NF-κB–driven metabolic reprogramming in CRC.^234^ A-hydroxybutyrate (α-HB), a metabolic byproduct elevated in CRC patients, induces the nuclear translocation of LDHA. Upon nuclear translocation, LDHA interacts with phosphorylated p65, thereby prolonging NF-κB retention and amplifying inflammatory signaling. This process can be further reinforced by oxidative stress (ROS), which also promotes LDHA nuclear localization, suggesting a positive feedback loop that stabilizes NF-κB activity. Although the study did not directly quantify metabolic fluxes such as glycolytic rate or ATP production, these findings reveal a novel mechanism by which α-HB links altered metabolism to NF-κB nuclear dynamics and CRC progression.^235^ Mechanistic insights also come from experimental models: a recent study using CT26 colon cancer cells demonstrated that RelA directly sustains mitochondrial function under metabolic stress by regulating the p53–synthesis of cytochrome c oxidase 2 (SCO2) axis. Loss of RelA disrupted OXPHOS and increased necrosis, underscoring its critical role in maintaining metabolic homeostasis under stress.^236^ These findings highlight the dual, context-dependent role of NF-κB: acting as a metabolic “guardian” under stress while also promoting tumor progression. Although these observations were primarily derived from CT26 models, they provide important mechanistic clues for understanding NF-κB-driven metabolic adaptation in CRC and warrant further validation in human studies. More recently, another report revealed that Emodin, a natural anthraquinone derivative, induces ferroptosis in CRC cells (HCT-15, SW620, HT-29, and RKO) by promoting nuclear receptor coactivator 4 (NCOA4)-mediated ferritinophagy and suppressing NF-κB signaling. Chemical induction of NF-κB by PMA partially rescued ferroptosis, confirming that NF-κB inactivation contributes to the metabolic vulnerability induced by Emodin.^237^ Nevertheless, the metabolic consequences of non-classical NF-κB signaling remain poorly understood.

#### *Metabolic reprogramming in CRC with Ras mutations*

The RAS oncogene family member, discovered over 40 years ago, is one of the earliest identified cancer initiation genes.^238^ Humans have three RAS genes: KRAS, NRAS, and HRAS, which give rise to four distinct RAS proteins belonging to the small GTPase family. As binary switches, these proteins play a critical role in cell signaling. Mutations that activate RAS are one of the most prevalent drivers of oncogenesis in human cancers, with KRAS standing out as the most often mutated oncogene.^239^

#### *KRAS and glutamine dependency*

CRC is often driven by sequential mutations in APC and KRAS, which stimulate cell growth and enhance invasion and spread. Wong et al observed that KRAS mutations created a reliance on glutamine, and its deficiency induced apoptosis in 1CT–AK cells. Compared with genetically identical normal cells, KRAS mutant CRC cells demonstrated significantly increased glutamine uptake. Additionally, the presence of succinate can restore growth in the absence of glutamine, as KRAS facilitated the conversion of glutamine to α-KG through SLC25A22, thereby enhancing TCA cycle intermediates, particularly succinate. The accumulation of succinate was linked to increased DNA methylation, WNT pathway activation, elevated LGR5 expression, and resistance to 5-FU.^240^ In line with these findings, clinical and experimental evidence has revealed that KRAS mutations also up-regulate the glutamine transporter SLC1A5, further reinforcing glutamine dependency in CRC. High SLC1A5 expression correlates with poor prognosis, and its knockdown markedly impairs the growth of KRAS-mutant CRC cells, underscoring its role as a metabolic driver and potential therapeutic target.^101^

#### *Glycolysis versus OXPHOS in KRAS-mutant CRC*

Early functional and proteomic studies consistently demonstrate that oncogenic KRAS reprograms central carbon metabolism in CRC, yet the degree and nature of these alterations are strongly allele- and context-dependent. For instance, under glucose limitation, KRAS mutations promoted stable GLUT1 up-regulation and a glycolytic phenotype, with KRAS^G12D^ being the most frequently detected mutation among surviving clones, whereas proteomic profiling of KRAS^G13D^ models revealed reproducible but comparatively modest up-regulation of glycolytic and anaplerotic enzymes.^241^^,^^242^ Similarly, KRAS^G12V^ cells display enhanced glycolysis, elevated lactate production, and marked sensitivity to glycolytic inhibition; mathematical modeling confirmed that this dependency is critically sustained by MCT1-mediated lactate transport.^243^ By contrast, the influence of KRAS mutations on OXPHOS appears more dynamic across disease stages. Rebane-Klemm et al reported that KRAS-mutant adenomas exhibited elevated Vmax of ADP-activated respiration and increased mitochondrial outer membrane permeability. However, in advanced KRAS-mutant CRC, OXPHOS capacity was partially reduced, with Vmax values lower than in KRAS wild-type tumors but still higher than adjacent normal mucosa.^244^ Together, these findings underscore that KRAS-driven metabolic rewiring cannot be generalized as a uniform “Warburg effect”. Instead, allele identity and lesion stage jointly shape the glycolytic–oxidative equilibrium, with critical implications for timing and context of metabolism-targeted therapies. A structured overview of these allele- and stage-specific metabolic features is summarized in Table 1 to highlight their mechanistic diversity and therapeutic relevance.

**Table 1:** Allele- and stage-specific metabolic reprogramming driven by KRAS mutations in colorectal cancer.

KRAS mutation type	Metabolic alteration	Method	Key findings/Therapeutic relevance
KRAS^G12D^	Strong glycolytic shift; increased glucose uptake and lactate production	Glucose limitation selection assays; GLUT1 expression analysis	KRAS^G12D^ was the most frequent mutation enriched under nutrient stress, driving stable GLUT1 upregulation and glycolytic dependency; predicts sensitivity to glycolytic inhibitors.
KRAS^G13D^	Modest upregulation of glycolysis, PPP, and amino acid metabolism	Proteomic profiling (cell models and patient samples)	Metabolic rewiring is reproducible but less pronounced than KRAS^G12D^; phenotype not proportional to KRAS abundance; suggests allele- and context-specific heterogeneity.
KRAS^G12V^	Strong glycolytic shift with high lactate output	Cell line experiments, metabolic flux assays, mathematical modeling	Cells highly sensitive to direct glycolysis inhibition (*e.g.*, BrPy, L-GA). MCT1 critical for lactate handling, but single-agent MCT1 inhibition ineffective due to MCT4 compensation. Therapeutic implication: Prioritize glycolysis-targeted or combination strategies rather than MCT1 monotherapy.
KRAS-mutant (stage-dependent)	Altered OXPHOS capacity	High-resolution respirometry of patient-derived CRC and adenoma tissues; assessment of ADP-activated respiration (Vmax) and mitochondrial outer membrane (MOM) permeability	KRAS-mutant adenomas: elevated Vmax and increased MOM permeability. Advanced KRAS-mutant CRC: Partially reduced OXPHOS capacity, with Vmax lower than KRAS-WT tumors but still higher than adjacent normal mucosa. Highlights stage-specific mitochondrial adaptations that may influence therapy responses.

#### *Amino acid metabolism and stress adaptation*

In fact, eight years ago, a study by Kosuke Toda and colleagues established that KRAS mutations modified amino acid metabolism in CRC by increasing asparagine levels. They found that these mutations up-regulated ASNS expression via the PI3K–AKT–mTOR cascade. CRC cells with KRAS mutations adapted to a lack of glutamine by synthesizing asparagine.^245^ Aligning with these observations, Najumudeen et al demonstrated that concomitant APC and KRAS mutations in the intestinal epithelium profoundly rewire metabolism, including increased glutamine consumption. Under this nutrient stress and KRAS activation, the system-L amino-acid antiporter SLC7A5 (LAT1) is transcriptionally and metabolically induced to import essential amino acids (notably leucine), thereby maintaining intracellular amino-acid pools and sustaining mTORC1 signaling. Genetic suppression of *Slc7a5* disrupted amino-acid homeostasis after KRAS activation and impaired both tumor initiation and late metastatic fitness, establishing SLC7A5 as a bona fide stress-adaptation node in KRAS-mutant CRC.^246^

#### *Therapeutic opportunities: MCT1, SLC1A5, SLC25A22, SLC7A5, and KRAS*^*G12C*^*inhibitors*

In KRAS-mutant CRC, the reliance of tumor cells on altered metabolic pathways has led to the identification of several promising therapeutic targets. MCT1, a key lactate transporter involved in sustaining aerobic glycolysis, has emerged as a viable target. AZD3965, a selective small-molecule inhibitor of MCT1, has completed a phase I dose-escalation clinical trial in patients with advanced solid tumors, including CRC. The study demonstrated favorable safety and target engagement profiles.^247^ Future investigations should focus on evaluating the efficacy of AZD3965 in tumors with high MCT1 but low MCT4 expression, particularly in metabolically driven CRC subtypes.

SLC1A5 (ASCT2) has also gained attention as a crucial glutamine transporter in KRAS-driven metabolic reprogramming. Among the inhibitors developed to date, MEDI7247, an antibody–drug conjugate (ADC) targeting ASCT2, has advanced into a phase I clinical trial in patients with relapsed or refractory hematologic malignancies.^248^ Although its application in solid tumors such as CRC has not yet been evaluated, this early clinical progress underscores the growing translational interest in targeting glutamine transport pathways in cancer metabolism.

Another mitochondrial glutamate transporter, SLC25A22, has been implicated in metabolic reprogramming in KRAS-mutant CRC. Preclinical studies have shown that genetic ablation of SLC25A22 markedly reduces resistance to 5-FU, inhibits tumor growth, and decreases the proportion of cancer stem-like cells.^240^ Although specific small-molecule inhibitors of SLC25A22 have not yet entered clinical development, recent findings highlight its clinical promise for KRAS-driven CRC, particularly through its roles in metabolic regulation, epigenetic remodeling, and immune evasion.

Extending the stress-adaptation theme, Najumudeen et al reported that *Slc7a5* loss rendered KRAS-mutant intestinal models responsive to rapalogs. Rapamycin suppressed proliferation in *Slc7a5*-deficient organoids and prolonged symptom-free survival in *Apc*^*fl/+*^;*Kras*^*G12D/+*^;*Slc7a5*^*fl/fl*^ mice; everolimus likewise curtailed tumor growth in Slc7a5-deficient tumors, whereas *Apc*^*fl/+*^;*Kras*^*G12D/+*^ tumors remained insensitive to mTOR inhibition. These findings position LAT1 loss as a context that sensitizes KRAS-driven CRC to mTORC1 blockade, providing a rationale to test LAT1 inhibition alone or in combination with rapalogs in KRAS-mutant CRC.^246^ From a translational standpoint, the selective LAT1 inhibitor JPH203 has undergone Phase I evaluation in biliary tract cancer, showing feasibility, tolerability, and preliminary activity.^249^ While CRC-specific trials have not yet been reported, these human data establish the clinical practicability of LAT1 targeting and, together with the preclinical rapalog-sensitization seen upon *Slc7a5* loss, support Phase I exploration in biomarker-enriched CRC cohorts (*e.g.*, LAT1-high, KRAS-mutant), including combinations with mTORC1 inhibitors.

While efforts to develop direct KRAS inhibitors have recently advanced, their applicability remains limited to specific mutation subtypes. KRAS^G12C^ inhibitors, such as sotorasib and adagrasib, have shown clinical benefit and are currently being tested in combination with EGFR-targeted agents in CRC. In contrast, KRAS^G12V^—one of the more frequent mutations—remains a therapeutically challenging target. No selective inhibitors have reached clinical use, and current strategies are focused on indirect targeting through metabolic dependencies or rational drug combinations.^250^^,^^251^

#### *KRAS as a metabolic hub in CRC: Targeting metabolic vulnerabilities to overcome therapy resistance*

Emerging studies highlight oncogenic KRAS as a central metabolic driver in CRC, influencing glycolysis, mitochondrial function, and energy sensing to facilitate tumor growth and resistance to treatment. KRAS-driven MAPK signaling phosphorylates tripartite motif-containing protein 21 (TRIM21), disrupting its interaction with c-Myc and preventing its degradation through autophagy. This stabilization of c-Myc increases enolase 2 (ENO2) expression and glycolysis, contributing to regorafenib resistance. Restoring TRIM21 function with vilazodone reverses this process and resensitizes tumors to therapy, underscoring KRAS’s role in regulating proteostasis and metabolic flexibility.^252^ Additionally, in hypoxic conditions, KRAS-mutant CRC cells create a feedback loop with HIF-1α, enhancing glycolysis and impairing mitochondrial function. This is driven by the up-regulation of PDK1 and GLUT1, which inhibit PDH and disrupt the TCA cycle. Vitamin C can break this cycle by promoting prolyl hydroxylation of HIF-1α, leading to PDH reactivation, mitochondrial dysfunction, ATP depletion, and increased cell death, presenting a potential therapeutic approach.^253^ KRAS also interacts with energy-sensing pathways, where high glycolytic ATP suppresses AMPK activity, reducing apoptosis and conferring resistance to anti-EGFR therapies like cetuximab. However, activating AMPK with metformin can reverse this resistance.^254^^,^^255^ These findings demonstrate how KRAS governs metabolic changes and integrates various cellular signals, creating therapeutic opportunities by targeting its metabolic dependencies in CRC.

#### *Metabolic reprogramming in CRC with BRAF*^*V600E mutations*^

The RAF–MEK–ERK cascade is an extensively studied MAPK cascade involved in regulating cell growth. This pathway includes three RAF isoforms—ARAF, BRAF, and CRAF—along with their downstream kinases MEK1/2 and ERK1/2, forming a network that governs various physiological processes. Genetic changes within this pathway are highly common in human cancers and include several key mutations, such as BRAF^V600E^.^256^ Moreover, BRAF mutations were found to be more prevalent in females and in the proximal colon.^257^ The presence of these mutations contributes to significant metabolic reprogramming within cancer cells. KRAS and BRAF mutations-driven metabolic shift results in protein changes associated with glycolysis, serine synthesis, the non-oxidative PPP, and glutamine utilization. Common alterations were observed in cells with KRAS and BRAF mutations, as well as in colon cancers with KRAS mutations. In RKO cells, BRAF^V600E^ protein levels are associated with the degree of the Warburg effect and changes in protein abundance, whereas these attributes do not correlate with KRAS^G13D^ levels in DLD-1 cells.^242^ Consistent with a glycolytic bias, Padder et al reported that BRAF^V600E^ CRC cells exhibit dynamin-related protein 1 (DRP1)-mediated mitochondrial fragmentation; notably, PDK1 suppression reduced DRP1 activation and glycolytic output and curtailed proliferation, clonogenicity, invasion, and epithelial–mesenchymal transition markers, indicating a therapeutically exploitable metabolic–mitochondrial vulnerability.^258^ Complementing the cell-based evidence, *ex vivo* analysis of resected CRC and polyps found that BRAF-mutated samples exhibited reduced mitochondrial respiration relative to wild-type tumors and changes in the apparent mitochondrial outer-membrane permeability, aligning with a shift toward glycolysis.^244^ In addition, BRAF mutations occur early in serrated CRC, with Rzasa et al reporting that BRAF^V600E^ disrupts intestinal epithelial cell homeostasis and impairs enterocyte differentiation over time. This mutation leads to persistent transcriptional changes linked to proliferation and metabolic reprogramming, as well as increased cholesterol biosynthesis in human lesions. Atorvastatin has been shown to prevent intestinal crypt hyperplasia in BRAF^V600E^ mutant mice, suggesting its potential in addressing CRC associated with BRAF mutations.^259^ On balance, available evidence shows that BRAF^V600E^ CRC integrates a DRP1–PDK1-driven glycolytic shift with early mevalonate–cholesterol pathway activation, revealing targetable metabolic liabilities beyond MAPK control. Given limited durability with current BRAF–EGFR/MEK regimens and immunotherapy, biomarker-guided co-targeting of PDK1/mitochondrial dynamics or the mevalonate axis merits formal testing.

#### *Metabolic characteristics of consensus molecular subtypes (CMS) in CRC*

In CRC, the consensus molecular subtypes (CMS1–4) offer a robust framework for stratifying tumors anchored in discriminative biomolecular and phenotypic profiles. CMS1 is classified as an MSI immune subtype, marked by substantial immune cell infiltration and activation. CMS2, on the other hand, is characterized by significant activation of the WNT and MYC signaling pathways.^260^ In contrast, mesenchymal consensus molecular subtype 4 (CMS4) is defined by mesenchymal features, poor prognosis, and increased stromal infiltration. A key metabolic feature of CMS4 tumors is the dysregulation of lipid metabolism, which contributes to their aggressive phenotype. Recent studies have shown that CMS4 tumors display up-regulation of lipolytic enzymes such as carboxylesterase 1 (CES1), regulated by transcription factors like hepatocyte nuclear factor 4 alpha (HNF4A) and NF-κB. CES1 catalyzes the hydrolysis of intracellular triacylglycerols, promoting FAO and OXPHOS, particularly under nutrient-limiting conditions. This metabolic shift not only supports cellular energy production but also prevents the accumulation of toxic lipids, protecting tumor cells from apoptosis and ferroptosis. Additionally, CES1 may facilitate metabolic crosstalk between CRC cells and adipocytes in fat-rich microenvironments, further supporting tumor progression and metastatic spread. These findings highlight the reliance of CMS4 tumors on lipid catabolism and suggest that targeting CES1 or FAO pathways could offer a promising therapeutic approach.^261^ In addition, CMS3 is recognized as the metabolic subtype of CRC, exhibiting alterations in both glucose and lipid metabolism. Emerging research indicates that CMS classification can be extended to metastatic CRC, revealing subtype-specific patterns of metastasis and metabolic adaptation. For example, CMS3 tumors are more frequently observed in brain metastases derived from rectal cancers, whereas CMS1 tumors are more common in liver metastases originating from right-sided colon tumors. Notably, CMS3 brain metastases display distinct metabolic signatures compared with their primary counterparts, with activation of OXPHOS, aerobic glycolysis, and lipid oxidation pathways. While CMS subtypes are prognostic in liver metastases, no significant survival differences have been observed across CMS groups in brain metastases. However, stratification based on the expression of CMS3-associated metabolic genes, such as PIK3R3, RET, CA8, and UGT8, identified subgroups with significantly poorer survival. These findings suggest that metabolic gene signatures may provide superior prognostic value over CMS classification alone in brain metastasis settings.^262^ These results emphasize the need for further investigation into the metabolic vulnerabilities of CRC subtypes, which could enhance therapeutic precision for both primary and metastatic CRC.

#### *Metabolic reprogramming in CRC metastasis*

In CRC patients, 90% die due to metastasis.^263^ Liver metastasis from CRC is a major contributor to mortality associated with cancer.^264^ Aldolase B (ALDOB), which is present in the intestine and associated with glycolysis, is up-regulated in CRC liver metastases.^265^ Alexander Nguyen and his team conducted a large-scale RNAi screening to uncover genes essential for the ability of CRC cells to invade the liver. They discovered that the isoform of pyruvate kinase expressed in the liver and red blood cells (PKLR) facilitates the metastatic colonization of these cancer cells. PKLR aids in the survival of malignant cells in environments marked by elevated cell density and low oxygen levels, typical of the liver microenvironment, which includes hypoxic areas and intense metabolic activity. Under these conditions, PKLR down-regulates the glycolytic role of the principal pyruvate kinase isoform, PKM2, which can be inhibited by ROS. This regulation supports cellular antioxidant responses, thereby maintaining levels of glutathione—an essential antioxidant—and improving the survival of cancer cells.^266^^,^^267^ Beyond PKLR-linked glycolytic/redox adaptation, CRC liver metastases also engage a cholesterol-specific adaptation: hepatocyte HGF triggers c-Met/PI3K/AKT/mTORC, selectively elevating SREBP2-driven cholesterol synthesis in hepatic lesions. As summarized in the “Potential Clinical Applications of Lipid Metabolic Biomarkers” subsection, this organ-selective induction nominates cholesterol biosynthesis as a tractable liver-specific target.^130^ Additionally, Zhang et al revealed that exosomes originating from CRC cells are crucial in creating a pre-metastatic microenvironment that facilitates metastasis to the liver. Exosome HSPC111 enhances the fatty acid metabolism of hepatic stellate cell-derived CAFs by phosphorylating ACLY, which increases acetyl-CoA levels. This elevation in acetyl-CoA promotes C-X-C motif chemokine ligand 5 (CXCL5) secretion from CAFs, driving epithelial–mesenchymal transition and CRC dissemination via the CXCL5–CXCR2 axis.^118^ From a therapeutic standpoint, targeting HSPC111 or its downstream fatty acid metabolic pathway may offer a promising strategy to disrupt CRC liver metastasis. Importantly, while Zhang et al focused on stromal metabolic remodeling, complementary work has demonstrated the immunological consequences of this axis: in CRC liver metastasis with the background of nonalcoholic fatty liver disease, CXCL5 secreted from Kupffer cells recruits CXCR2^+^ myeloid-derived suppressor cells, establishing an immunosuppressive niche and rendering tumors refractory to anti-PD-1 therapy. Notably, pharmacological blockade of myeloid-derived suppressor cell recruitment with Reparixin restored CD8^+^ T cell infiltration, reversed immune checkpoint inhibitor resistance, and markedly inhibited CRC liver metastases in preclinical models.^268^ Collectively, these findings highlight not only the dual stromal–immune reprogramming mediated by exosomes and chemokines but also point to Reparixin—already pharmaceutically available—as a promising candidate for rapid clinical translation to overcome immune checkpoint inhibitor resistance in colorectal liver metastasis. In addition to liver metastasis, approximately 4% of CRC patients experience ovarian metastasis.^269^ Single-cell and spatial profiling have shown that the ovarian metastatic niche of CRC (oCRC) contains a myofibroblast subset (RBP1^+^) that is enriched specifically in oCRC. Metabolite–sensor analysis indicated dominant l-glutamine communications from these myofibroblasts to farnesyl diphosphate synthase-positive (FDPS^+^) malignant cells; concordantly, RBP1^+^ myofibroblasts expressed high glutamate-ammonia ligase (GLUL), whereas FDPS^+^ tumor cells expressed lower GLUL, consistent with reliance on exogenous glutamine. FDPS^+^ cells also exhibited heightened OXPHOS, glycolysis, d-glutamine/d-glutamate metabolism, and TCA-cycle activity. Functionally, conditioned medium from human ovarian fibroblasts increased intracellular glutamine and proliferation of CRC cells, effects attenuated by the ASCT2 (SLC1A5) inhibitor V-9302.^4^ These data position oCRC as a niche in which RBP1^+^ myofibroblasts supply glutamine (high GLUL) to FDPS^+^ malignant cells with heightened OXPHOS/glycolysis/TCA activity. Going forward, ^13^C-glutamine tracing in patient-derived organoids co-cultures with genetic perturbation of GLUL/SLC1A5, spatial metabolomics, and pharmacologic testing of LAT1/SLC1A5 and FDPS/mevalonate blockade will be critical to establish causality and druggability. A composite biomarker (RBP1^+^ stroma + stromal GLUL + tumor SLC1A5/FDPS) and exploratory trials integrating SLC1A5 inhibition with standard regimens could translate this stromal–tumor metabolic coupling into oCRC-specific therapeutic strategies.

## Conclusion and perspectives

Over the past few decades, rewiring of bioenergetic networks has been established as a key factor in tumor initiation, progression, and prognosis. Tumor cells, by reshaping metabolic pathways, not only adapt to the harsh tumor microenvironment but also influence various cell populations within it, including lymphocytes and fibroblasts, thereby promoting immune evasion, tumor invasion, and metastasis. Metabolic reprogramming in CRC, in particular, has garnered increasing attention as a crucial factor influencing tumor behavior.

Currently, the increasing incidence in younger patients and the emergence of treatment resistance in colorectal tumors are of great concern, making the exploration of innovative early diagnostic markers and therapeutic strategies particularly important.^270^ Although significant advancements have been achieved in the last few years regarding metabolic reprogramming in CRC, several key challenges remain. First, while metabolic reprogramming is widely acknowledged as a crucial feature of cancer, our understanding of its precise mechanisms, particularly in the context of different CRC stages and tumor types, remains incomplete. Many studies remain in their early stages, and the complex relationship between metabolic pathways and tumor biology remains incompletely deciphered. Second, the role of metabolic rewiring in the tumor microenvironment remains underexplored, especially regarding how immune cells, fibroblasts, and other non-tumor cells regulate immune evasion and metastasis. This area requires further experimental validation. In parallel, research on CRC stem cell metabolism is still in its infancy, with a lack of well-established experimental models and clinical data supporting their role in drug resistance and relapse. Moreover, the study of metabolic reprogramming in MSS CRC subtypes has been limited, and a critical knowledge deficit persists regarding the metabolic adaptations that drive tumor progression in these patients. MSS CRC, which accounts for the majority of CRC cases, has not been extensively studied in terms of its metabolic characteristics or their influence on therapy responses, particularly with regard to immune checkpoint inhibitors. The paucity of metabolic research in MSS CRC underscores the need for targeted studies that can elucidate the metabolic vulnerabilities of this more common CRC subtype, with potential implications toward engineering novel precision interventions and enhancing clinical trajectories.

In conclusion, metabolic reprogramming plays a central role not only in tumor cell survival and proliferation but also in mediating complex phenomena such as immune evasion and metastasis. Subsequent investigations ought to delineate the molecular logic orchestrating CRC stem cell metabolism, exploring how these pathways influence tumor initiation, resistance, and relapse. Additionally, addressing the research gaps in the metabolic characterization of MSS CRC will be essential for advancing understanding and developing targeted therapies. By improving our understanding of metabolic rewiring in CRC, we can identify new therapeutic targets and strategies, potentially enhancing treatment efficacy and patient prognosis.

## CRediT authorship contribution statement

**Wen Xiao:** Writing – review & editing, Writing – original draft. **Dan Tan:** Conceptualization. **Wen Zeng:** Supervision. **Xiaoming Nie:** Visualization. **Mingliang Wang:** Writing – review & editing, Project administration, Funding acquisition.

## Funding

This work was supported by the 10.13039/501100001809National Natural Science Foundation of China (No. 82272633) and 10.13039/501100004479Jiangxi Provincial Natural Science Foundation (China) (No. 20242BAB20426).

## Conflict of interests

The authors declare that they have no known competing financial interests or personal relationships that could have appeared to influence the work reported in this paper.
